# Coordinated Regulation of Rsd and RMF for Simultaneous Hibernation of Transcription Apparatus and Translation Machinery in Stationary-Phase *Escherichia coli*

**DOI:** 10.3389/fgene.2019.01153

**Published:** 2019-12-04

**Authors:** Hideji Yoshida, Akira Wada, Tomohiro Shimada, Yasushi Maki, Akira Ishihama

**Affiliations:** ^1^Department of Physics, Osaka Medical College, Takatsuki, Japan; ^2^Yoshida Biological Laboratory, Kyoto, Japan; ^3^School of Agriculture, Meiji University, Kawasaki, Japan; ^4^Research Center for Micro-Nano Technology, Hosei University, Koganei, Japan

**Keywords:** RNA polymerase sigma factor, anti-sigma factor (Rsd), ribosome, ribosome modulation factor, hibernation, stationary phase, Escherichia coli K-12

## Abstract

Transcription and translation in growing phase of *Escherichia coli*, the best-studied model prokaryote, are coupled and regulated in coordinate fashion. Accordingly, the growth rate-dependent control of the synthesis of RNA polymerase (RNAP) core enzyme (the core component of transcription apparatus) and ribosomes (the core component of translation machinery) is tightly coordinated to keep the relative level of transcription apparatus and translation machinery constant for effective and efficient utilization of resources and energy. Upon entry into the stationary phase, transcription apparatus is modulated by replacing RNAP core-associated sigma (promoter recognition subunit) from growth-related RpoD to stationary-phase-specific RpoS. The anti-sigma factor Rsd participates for the efficient replacement of sigma, and the unused RpoD is stored silent as Rsd–RpoD complex. On the other hand, functional 70S ribosome is transformed into inactive 100S dimer by two regulators, ribosome modulation factor (RMF) and hibernation promoting factor (HPF). In this review article, we overview how we found these factors and what we know about the molecular mechanisms for silencing transcription apparatus and translation machinery by these factors. In addition, we provide our recent findings of promoter-specific transcription factor (PS-TF) screening of the transcription factors involved in regulation of the *rsd* and *rmf* genes. Results altogether indicate the coordinated regulation of Rsd and RMF for simultaneous hibernation of transcription apparatus and translation machinery.

## Introduction

Batch cultures under optimal laboratory conditions of the well-characterized model bacterium *Escherichia coli* in rich media at an optimum temperature (usually at 37°C, the temperature of host animals for enterobacterium *E. coli*) under sufficient supply of oxygen exhibit a progression of constant steady-state growth as measured by either counting of the viable cells or measuring the cell turbidity. Traditionally, the cell growth has been classified into three phases: non-replicative lag phase; replicative exponential phase; and stationary phase of replication cessation. The growing-phase *E. coli* has long been used as a model organism relying on the belief that its laboratory culture is homogenous in cell populations. Most of our knowledge of modern molecular genetics such as the mechanisms and regulation of gene expression was established using such apparently homogenous planktonic cell cultures.

In contrast to the laboratory culture conditions, the conditions that allow steady-state bacterial growth are seldom found in nature. Instead, the lack of nutrients, accumulation of toxic waste compounds, and the influence of harsh environmental conditions such as lack of oxygen and pH change threaten the survival of *E. coli*. A variety of protection systems against such hazardous environments are induced for survival by changing the cell organization at both the molecular and cellular levels (Foster, 1999; Raivio, 2005; Battesti et al., 2011; Jin et al., 2012; Mehta et al., 2015). Under such a background, the focus in *E. coli* research is being shifted toward understanding the survival strategy of *E. coli* after growth cessation. Facing this research stage, *E. coli* is again recognized as a suitable model organism because of huge amounts of accumulated knowledge of *E. coli* such as the functions and regulation of the whole set of genes on its genome.

**Figure 1:**
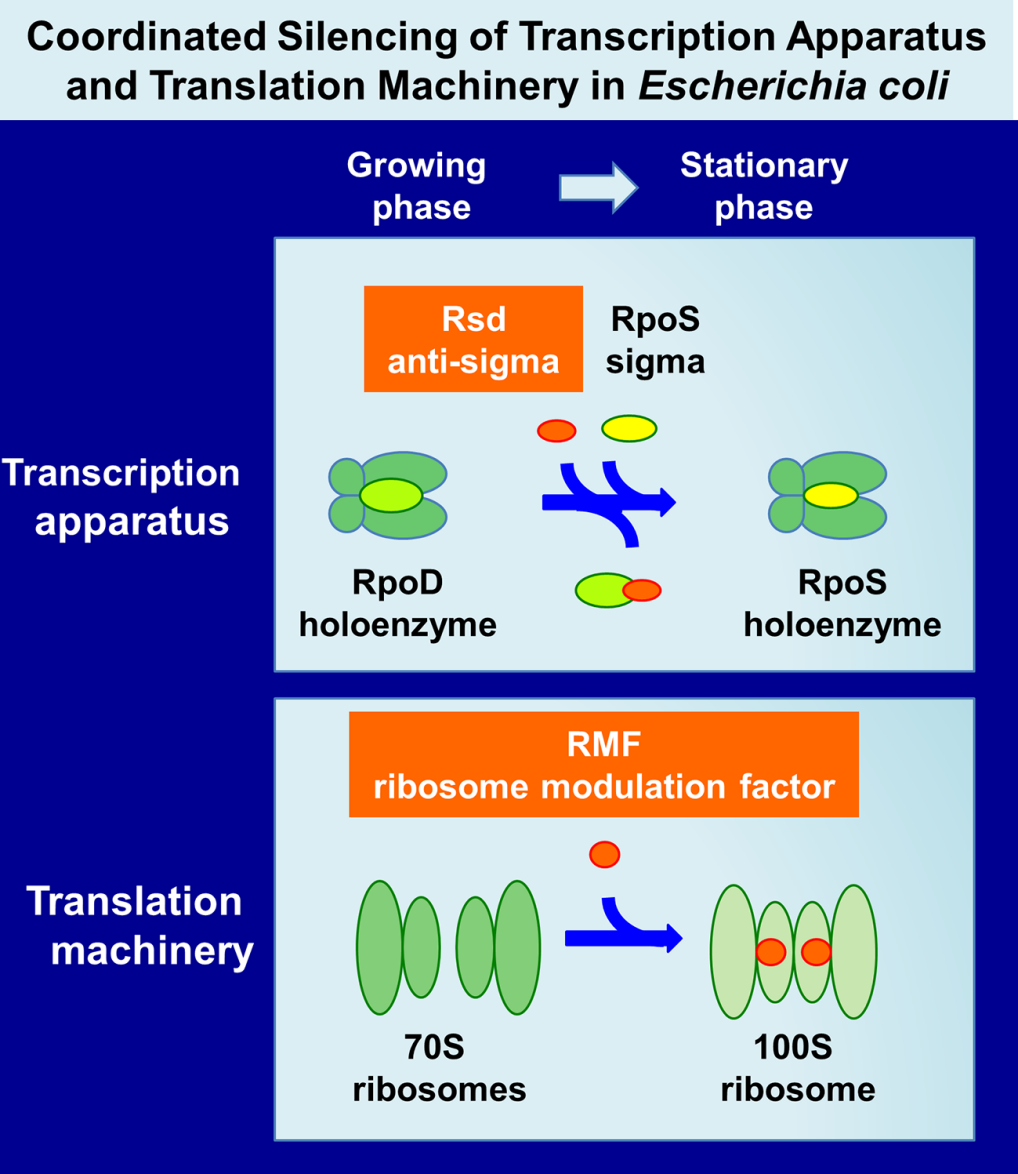
Hibernation of transcription apparatus and translation machinery in *Escherichia coli* K-12. Upon entry of *E. coli* growth into the stationary phase, RNAP RpoD becomes silent through binding of anti-sigma factor Rsd onto the RpoD region-4 (promoter -35 recognition site) (Jishage and Ishihama, 1998; Jishage et al., 2001) while functional 70S ribosomes are converted to inactive 100S dimers through association with RMF (Wada et al., 1990; Wada, 1998) and HPF (Ueta et al., 2008; Yoshida and Wada, 2014). Here, we describe the coordinated regulation of two key regulators, Rsd and RMF, in *E. coli* K-12. The binding targets and binding sites of these two regulators on RNAP and ribosomes are described in text and also in Figure 6. Other factors involved in these processes are also described in text. RNAP, RNA polymerase; RMF, ribosome modulation factor; HPF, hibernation promoting factor.

Upon entry into the stationary phase of laboratory *E. coli* cultures, a variety of morphological and physiological changes take place in individual cells. The growth phase-coupled changes in cell characteristics are associated with a change in expression pattern of the genome: most of the growth-related genes are turned off or leveled down, and, instead, a number of the genes needed for stationary-phase survival are expressed (for reviews, see Lowen and Hengge-Aronis, 1994; Ishihama, 1997; Ishihama, 1999). Overall level of genome expression decreased down to less than 10% of the level of exponential growth. The change in genome expression is mainly attributable to the changes in activity and specificity of gene expression system, including transcription apparatus and translation machinery in parallel with the structural reorganization of genome within the nucleoid (Figure 1). Upon entry into the stationary phase, unused excess cellular components are generally degraded for reuse as nutrients for survival. Both transcription apparatus and translational machinery are, however, stored without being degraded, and instead, their activity and specificity are markedly modulated for expression of the stationary-phase genes (referred to as “stationary genes” in this report). The major change of transcription apparatus is the replacement of the promoter-recognition subunit sigma from RpoD to RpoS through the aid of anti-sigma factor Rsd (regulator of sigma D) (Jishage and Ishihama, 1995) (Figure 1). On the other hand, 70S ribosome is converted into inactive 100S dimer with the aid of ribosome modulation factor (RMF) and hibernation promoting factor (HPF) (Maki et al., 2000; Ueta et al., 2005) (Figure 1). We found that these factors have been involved in detailed analyses of the regulatory roles of these factors (for reviews, see Wada, 1998; Ishihama, 1999; Ishihama, 2000a; Yoshida and Wada, 2014). Here, we provide an overview of the molecular basis of genome expression system after the stationary phase, focusing on the simultaneous and coordinated hibernation of the transcription apparatus and the translation machinery.

Up to the present time, a set of anti-sigma factors have been identified, each sequestering each of all seven *E. coli* K-12 sigma factors (Hughes and Mathee, 1998; Helmann, 1999; Trevino-Quintanilla et al., 2013; Paget, 2015). Similar systems of the functional modulation of RNA polymerase (RNAP) are also known in bacteria other than *E. coli*, but the knowledge of regulatory functions of the whole set of sigma and anti-sigma factors is best known for *E. coli* (for details, see *Hibernation of the Transcription Apparatus*). Likewise, the factors for ribosome silencing differ between *E. coli* and other bacteria. For instance, non-gamma proteobacteria form 100S ribosome but lack RMF and contain long HPF homologues (Ueta et al., 2008; Yoshida and Wada, 2014) (for details see *Hibernation of the Translation Machinery*). As to the silencing of transcriptional apparatus and translational machinery, we focus on the well-characterized *E. coli* K-12 systems in this review.

## Growth Phase-Coupled Changes in Cell Characteristics

### Discontinuous Change of the Cell Buoyant Density

Upon entry into the stationary phase of laboratory *Escherichia coli* cultures, a variety of morphological and physiological changes take place in individual cells, including decrease in cell size, alteration in cell shape, compaction of nucleoid, changes in cell wall organization, and alterations in cytoplasm compositions (Roszak and Colwell, 1987; Kolter et al., 1993; Huisman et al., 1996). The synchronization of cell growth is disturbed, supposedly due to difference in microenvironment, and accordingly, the stationary-phase culture includes a mixture of heterogeneous cell populations including dead cells. The level and mode of cell heterogeneity differ depending on the culture conditions or factors affecting growth retardation the (Ferenci, 2001; Stewart and Franklin, 2008; Martinez-Antonio et al., 2012; Serra and Hengge, 2014; Pletnev et al., 2015). Upon entry into the stationary phase, the cell wall becomes thicker while the cytoplasm becomes condensed. In parallel, a variety of changes have been recognized for the cell characteristics, including the increase of unsaturated fatty acids in membrane, the increase of osmoprotective solutes such as trehalose and glycine betaine in cytoplasm, the accumulation of storage compounds such as glycogen and polyphosphate, and the decrease in polyamines (Roszak and Colwell, 1987; Kolter et al., 1993; Huisman et al., 1996; Ishihama, 2000). The nucleoid becomes more compact by replacing the DNA-binding proteins, for instance, from Fis in the log-phase to Dps in the stationary phase (Talukder et al., 1999; Ishihama, 2009). The DNA superhelicity, however, decreases in the stationary phase (Jaworski et al., 1991; Kusano et al., 1996).

**Figure 2:**
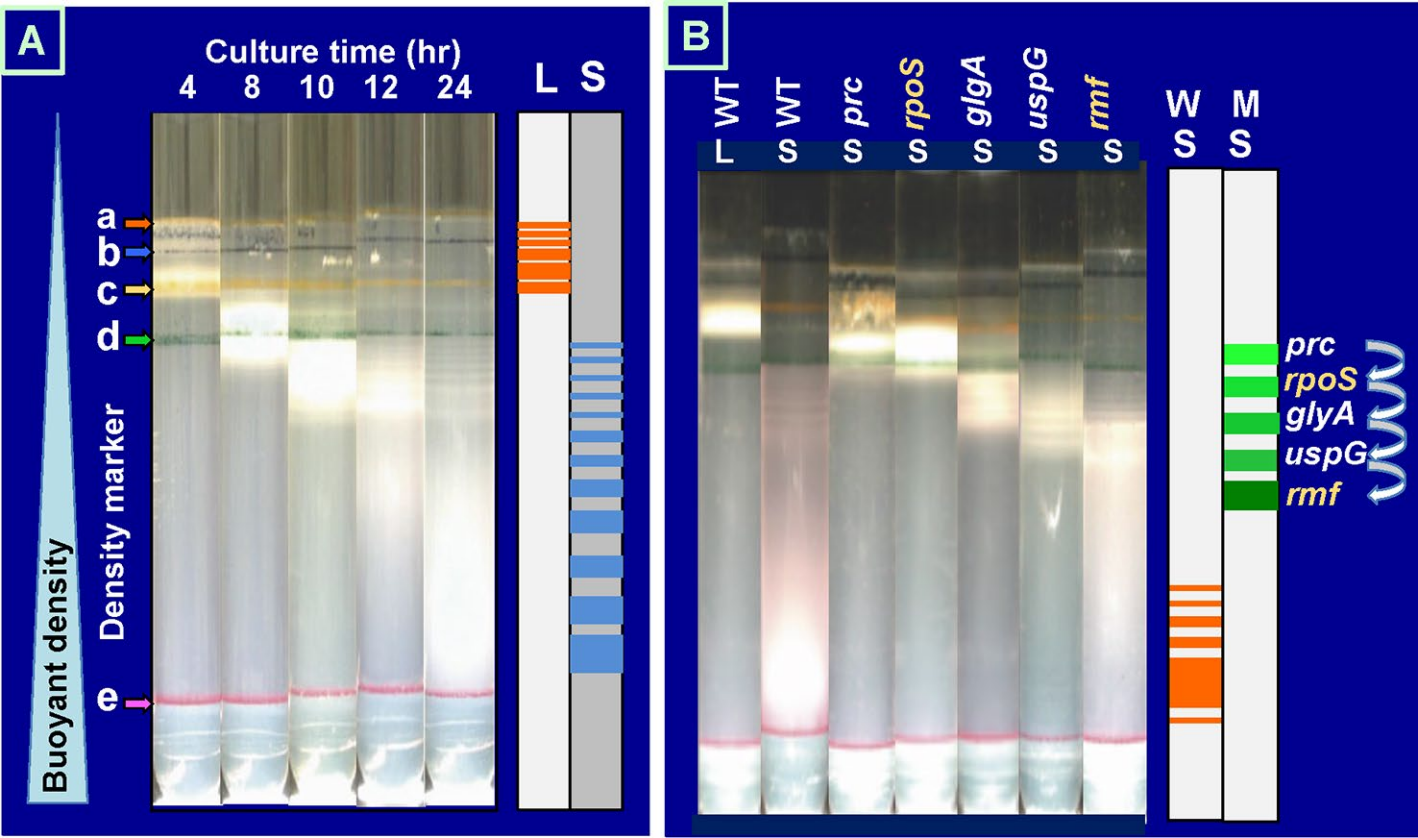
Growth phase-dependent discontinuous increase of cell buoyant density of *Escherichia coli* K-12. **(A)** *E. coli* W3110 was grown in LB medium at 37°C with shaking. At various times, an aliquot of cell suspension was subjected to Percoll gradient centrifugation for 1 h at 20,000 rpm at 4°C in a Beckman SW40Ti rotor (Makinoshima et al., 2002; Makinoshima et al., 2003). The location of marker beads is indicated on the left: a, 1.035 g/ml; b, 1.074 g/ml; c, 1.087 g/ml; d, 1.102 g/ml; e, 1.119 g/ml. **(B)***E. coli* wild-type BW25113 and its single-gene knockout mutants were grown in LB for 4 (L) or 24 h (S) and subjected to Percoll gradient centrifugation. The increase in cell buoyant density was interfered for these mutants, remaining at specific positions as indicated on the right. LB, lysogeny broth.

For physical separation of heterogeneous cell populations, we succeeded in separating *E. coli* cell populations using centrifugation through gradients of polyvinylpyrrolidone-coated silica Percoll that protects the cells from toxic effects of silica (Makinoshima et al., 2002; Makinoshima et al., 2003). Due to the low viscosity of Percoll, materials as large as marker beads and bacterial cells quickly sediment to positions characteristic of their densities. Exponential phase cultures of *E. coli* K-12 formed at least five discrete even though the density difference is within a narrow range (Figure 2A). This minor heterogeneity might correspond to the difference in the cycle of cell division (Kubitschek et al., 1983; Koch, 1996). In contrast, the stationary-phase cultures formed more than 10 bands, all exhibiting increased densities than the log-phase cultures (Figure 2A). A number of factors should influence the cell density, such as the cell volume, the chemical composition of cells, and the content of free water. One of the unexpected findings is the growth phase-coupled discontinuous transition of *E. coli* cell density. Even if the growth phase-coupled changes in molecular events are continuous, the overall cell characteristics change in discontinuous fashion as detected by the buoyant density. We concluded that the overall state of cell morphology and/or physiology of *E. coli* cells changes in discontinuous fashion during the growth transition from the log phase to the stationary phase.

**Figure 3:**
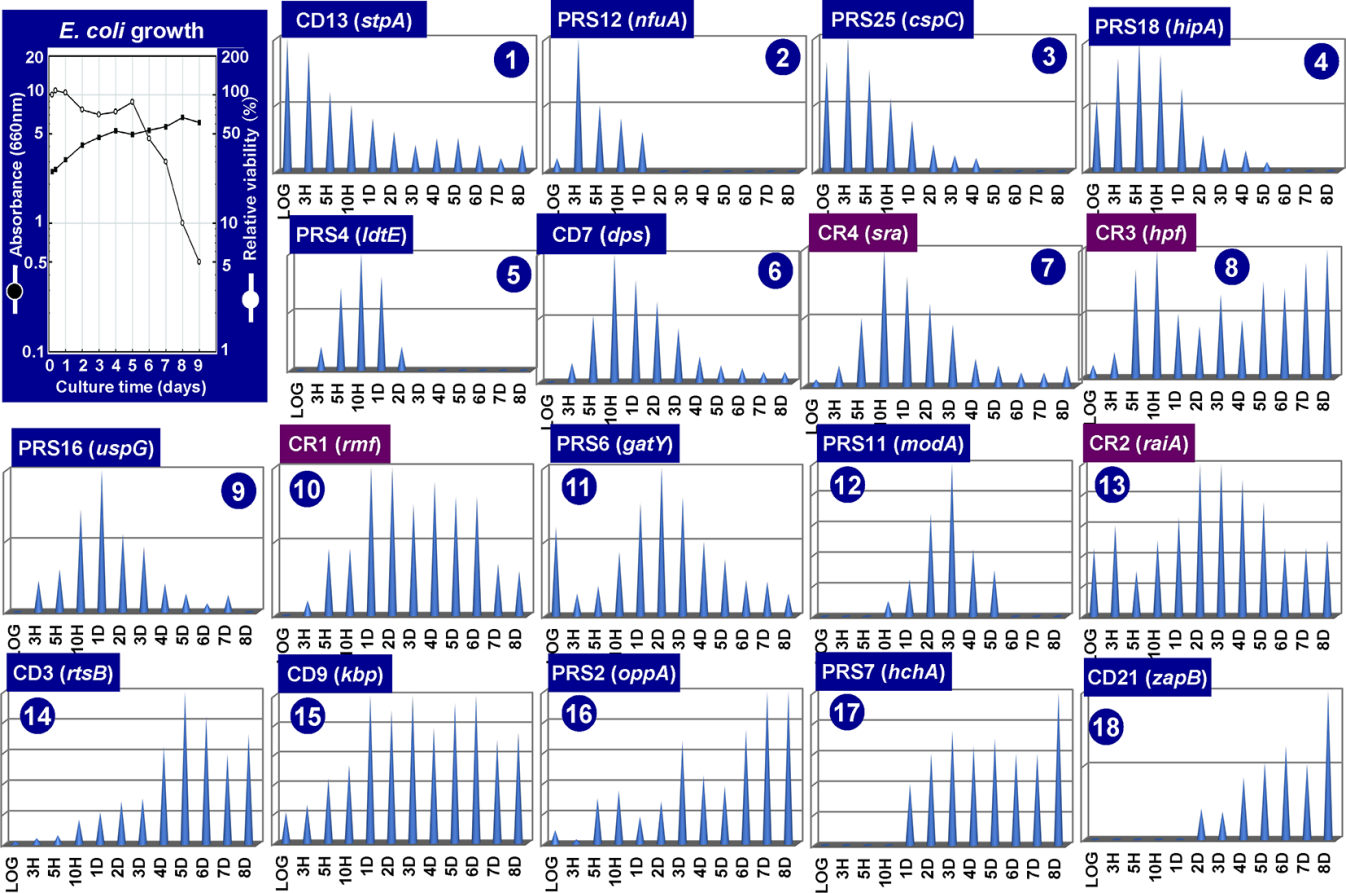
Growth phase-dependent synthesis of 18 representative stationary proteins in *Escherichia coli* K-12. *E. coli* K-12 AD202 was grown in minimal medium E (Vogel and Bonner, 1956) containing 2% peptone at 37°C. The cell growth was monitored for 10 days by measuring the turbidity at 660 nm and by counting viable cells as shown in the inset. Aliquots of the culture were harvested at the indicated time (X-axis), and the cell lysates were fractionated into CD (insoluble cell debris), CE (cell extract supernatant), CR (crude ribosome), and PRS (post ribosomal supernatant) fractions. All these fractions prepared at each time point was subjected to RFHR 2D gel system, and the stained protein spots were measured by densitometry. The relative levels (Y-axis) are shown at each culture time (X-axis) for a total of 18 representative stationary proteins. The proteins shown under purple background indicate those involved in the hibernation of ribosomes.

A number of stationary genes have been identified by transcriptome and proteome analyses (Franchini et al., 2015; Sanchuki et al., 2017; Caglar et al., 2018). At present, however, we have only fragmentary knowledge on the expression order and the physiological roles of these stationary genes. We realized that the discontinuous change in cell buoyant density is a good marker for identification of the genes involved in each step of the cell differentiation during the transition of cell growth from exponential to stationary phase. We then subjected more than 200 single-gene-knockout mutants from the Keio collection (Baba et al., 2006; Yamamoto et al., 2009) to Percoll gradient centrifugation. Some mutants exhibited altered distribution (see **Supplemental Figure S2** for protein distribution), mostly defective in the density increase even after prolonged centrifugation. For instance, the density increase was found to be impaired at an early step for a mutant *E. coli* with the disrupted *rpoS* gene, which encodes RpoS sigma, the key player of stationary gene transcription (**Figure 2B**). RpoS was found to be needed at the early stage of the cell density increase (for details, see next chapter). The interruption of density increase was observed for the genes not directly related to transcription. For instance, mutants defective in RpoF and RpoN exhibited essentially the same centrifugation pattern with that of wild-type *E. coli* K-12. In contrast, the density increase stopped for the mutant lacking the *rmf* gene at a step later than that for RpoS sigma, indicating that the ribosome dimerization takes place after expression of RpoS-dependent genes. Afterward, the density increase is interrupted for the mutant lacking universal stress protein (UspG) (**Figure 2B**). RMF is required for hibernation of ribosomes through conversion of functional 70S monomer to inactive 100S dimer (for details, see below) (Wada, 1998; Yoshida and Wada, 2014), while UspG is needed for cell–cell interaction in biofilm formation in the stationary phase (Nachin et al., 2005). The stop order of buoyant density increases for the *uspG* and *rmf* mutants agrees well with the order of maximum expression of UspG and RMF in wild-type *E. coli* (see **Figure 3**).

### Growth-Dependent Change of the Protein Expression Pattern

As noted above, the pattern of genome expression in the stationary-phase changes for adaptation and survival as measured by genome-wide expression patterns of mRNA and protein products using the modern omics systems. In this section, we focus on the expression and degradation of the whole set of stationary proteins during the prolonged culture after the stationary phase up to 8 days. For protein separation and identification, we employed the radical-free highly reducing (RFHR) system of two-dimensional (2D) gel electrophoresis (for details, see Wada, 1986a; Wada, 1986b). The RFHR method allowed fine resolution of proteins on 2D gels, minimizing artificial spots generated through intra-molecular and inter-molecular Cys–Cys bridging under oxidation circumstances. The level of each protein on the RFHR 2D gel pattern can be determined by measuring the density of stained protein spot (**Supplemental Figure S1**). For the analysis of stationary proteins, we used *E. coli* K-12 AD202 strain lacking the *ompT* gene encoding outer membrane protease 7, which exhibits strong protein hydrolysis activity during cell lysate preparation once liberated from the outer membrane. In the experiments shown in **Figure 3**, cells were harvested at various times up to day 8. Under the culture conditions employed, the viability decreased gradually to less than 10% at day 8 (**Figure 3**, inset). The whole cell lysates were fractionated by centrifugation into CD (insoluble cell debris) and CE (cell extract supernatant fraction), which were then fractionated into CR (crude ribosome fraction) and PRS (post ribosomal supernatant fraction) (for details, see **Figure 3** legend). The nature of each protein spot on RFHR 2D gel could be determined after protein sequencing and/or mass spectroscopy. After repeating RFHR analysis thoroughly, a total of more than 650 protein spots were identified, of which a total of 65 appeared or markedly increased after the stationary phase. These proteins were detected in three cellular fractions: 31 in RPS, 30 in CD, and 4 in CR (**Supplemental Table S1**). Up to the present time, a total of 48 spots have been identified, but 17 remained unidentified.

The RFHR system is in particular useful for analysis of small proteins, allowing the identification of these small-sized ribosome-associated proteins. The CR (crude ribosomal) fraction contained the newly identified 50S proteins, L35 (RpmI) and L36 (RpmJ) (Wada and Sako, 1987), and 30S protein S22 (Sra or RpsV) (Izutsu et al., 2001), leading to make the complete list of 54 r-proteins in *E. coli* K-12. Besides, some ribosome-related proteins were included in the CR fraction such as RMF, RaiA (renamed YfiA), and HPF (renamed YhbH), which all are involved in ribosome hibernation; for details, see *Hibernation of the Translation Machinery*.

The CD fraction recovered in the pellet fraction after low-speed centrifugation includes a total of 30 proteins tightly associated with cell wall and membrane. Stationary-phase-specific nucleoid proteins Dps and StpA were recovered in this CD fraction in agreement with the tight association of stationary-phase nucleoid with the cell membrane (Ishihama, 2009). Most of stress-response gene products in this CD fraction such as SlyD (chaperone with peptidyl-prolyl *cis-trans* isomerase activity) and StpA (H-NS-like nucleoid protein with RNA chaperone function), and two of six *E. coli* UspGs, UspD and UspG. All these proteins are involved in repair and refolding of RNAs and proteins (see **Supplemental Table S1**). The PRS fraction includes a total of 31 soluble stationary proteins, which all migrated in neutral to acidic regions on 2D (see **Supplemental Figure S1**). Most of these soluble proteins are involved in stationary-phase-specific metabolism, supposedly for redirection of metabolic circuits after prolonged culture in the absence of sufficient nutrients.

**Figure 4:**
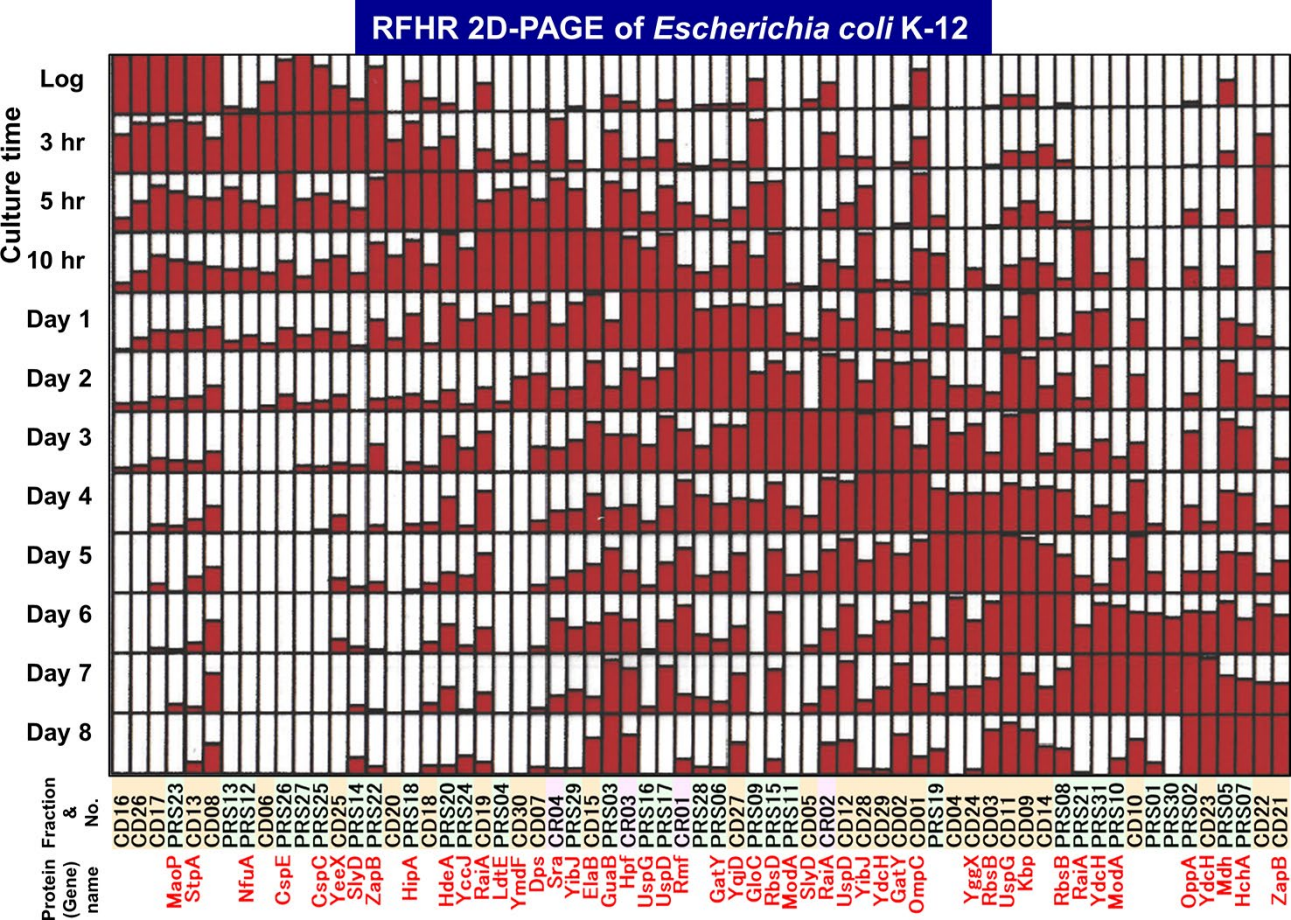
Growth phase-dependent expression patterns of a total of 65 stationary proteins in *Escherichia coli* K-12. The growth phase-dependent synthesis was measured for a total 65 stationary proteins. The relative level of synthesis from log phase (3-h culture) to day 8 is shown for all 65 proteins. The maximum level is shown by filling the day column with full red color. Spot numbers listed in **Table 1** are shown on the horizontal axis, with colors indicating fraction type (green: PRS, orange: CD, and magenta: CR). The protein products so far identified are shown in red below the corresponding spot numbers.

The level of stationary-phase proteins was measured throughout the culture up to day 8 (**Figure 3**), and the relative distribution is aligned in the order of appearance time throughout the 8-day culture (**Figure 4**). About half of the stationary-phase proteins appeared at specific time and soon disappeared, exhibiting a relatively narrow pattern of appearance in the stationary phase, but some other stationary proteins distributed in rather wide range of the stationary phase even though the distribution pattern between three subcellular fractions change. It should be noted that some stationary-phase proteins are detected in more than two fractions and exhibited culture time-dependent shift of distribution such as RPS-to-CD for GatY, RbsB, SlyD, UspD, ZapB, YdcH, and YibJ (see **Table 1**). The final deposition of these soluble proteins could be in the cell membrane and cell wall after prolonged culture. One exceptional distribution pattern was observed for RaiA, which showed a culture time-dependent alteration of distribution among all three fractions, CR, PRS, and CD (see **Table 1**), supposedly reflecting to its role in ribosome hibernation (see below).

**Table 1:** Proteins Expressed During Prolonged Culture of *Escherichia coli* K-12.

PRS		CD		CR						
2D spot	Max stage	2D spot	Max stage	2D spot	Max stage	Gene		Map	pI/Size (aa)	Function
**PRS16**	**Day 1**	**CD11**	**Log**			***uspG***	***ybdQ,yzzU***	13.79	6.03/142	universal stress protein G
**RPS26 **	**Late-log (3 h) **					***cspE***	***msmC***	14.16	8.09/69	transcription antiterminator/RNA stability regulator CspE
**PRS10/11 **	> **Day-3 Day-7 **					***modA***		17.12	7.81/257	periplasmic molybdate transporter protein
		**CD07**	**10 h**			***dps***	***pexB,vtm***	18.27	5.70/167	stationary-phase nucleoid protein/Fe-binding storage protein
**PRS09 **	**Day 3 **					***gloC***	***ycbL***	21.19	4.95/215	hydroxyacylglutathione hydrolase;methylglyoxal degradation
				**CR01**	**Day 1 and 2**	***rmf***		21.87	10.86/55	ribosome modulation factor
**PRS24 **	**Late-log (5 h) **					***yccJ***		22.97	4.70/75	PF13993 family protein YccJ
		**CD30**	**10 h**			***ymdF***		23.00	9.87/57	stress-induced acidphilic repreak motifs-containing protein
**RPS02 **	**Day 7 and 8 **					***oppA***		24.04	6.05/543	periplasmic oligopeptide transporter protein
**PRS31 **	**Day 2 **	**CD23**	**Day 7 and 8**			***ydcH***		32.29	9.30/74	uncharacterized protein
**PRS18 **	**Late-log (5 h) **					***hipA***		34.28	8.26/440	serine/threonine kinase HipA; regulator with hipB
				**CR04 **	**10 h **	***sra***	***rpsV***	35.52	11.04/45	30S ribosomal protein S22
**PRS04 **	**10 h **					***ldtE***	***ynhG***	37.87	9.42/334	L,D-transpeptidase
**PRS25 **	**Late-log (3 h) **					***cspC***	***msmB***	41.08	6.54/69	cold-shock stress protein CspC
**PRS07 **	**Day 8 **					***hchA***	***yedU,yzzC***	43.86	5.63/283	protein/nucleic acid deglycase; Hsp32 moleccular chaperone
		**CD25**	**Late-log (3 h)**			***yeeX***		44.79	9.30/109	DUF496 domain-containing protein
**PRS06 **	**Day 2 **	**CD02**	**Day 4**			***gatY***	***yegF***	46.91	5.87/284	tagarose-1,6-dibphosphate aldolase
		**CD01**	**Day 4**			***ompC***	***meoA,par***	49.82	4.58/367	outer membrane protein C pore for passive difusion
		**CD15**	**10 h and Day 1**			***elaB***	***yfbD***	51.34	5.35/101	tail-anchored inner membrane protein
**PRS03 **	**Day 7 **					***guaB***		56.60	6.02/486	Inosine 5’-monophosphate dehydrogenase; GMP symthesis GMP symthesis
**PRS21 **	**Day 7 **	**CD19**	**10 h**	**CR02 **	**Day 2 and 3 **	***raiA***	***yfiA***	58.88	6.19/113	stationary-phase translation inhibitor/ribosome stability factor
		**CD13**	**Log**			***stpA***	***hnsB,rsv***	60.19	7.95/134	nucleoid protein StpA with RNA chaperone activiry
		**CD09**	**Day 6**			***kbp***	***ygaU,yzzM***	60.24	5.67/149	K+ binding protein
		**CD24**	**Day 5**			***yggX***		66.78	5.91/91	Fe2+-tracking protein; oxidative damage protect Fe-S protein
		**CD27**	**Day 2**			***yqjD***		69.91	9.06/101	ribosome- and membrane-associated DUF-domain protein
				**CR03 **	**10 h**	***hpf***	***yhbH***	72.01	6.50/95	ribosome hibernation-promoting factor; RpoN modulation protein
**RPS05 **	**Day 8 **					***mdh***		72.81	5.61/312	malate dehydrogenase
**PRS22 **	**Late-log (3 and 5 h) **	**CD21**	**Day 8**			***zapB***	***yiiU***	75.71	4.69/81	cell division factor ZapB
**RPS17 **	**Day 1 **	**CD12**	**Day 3 and 7**			***uspD***	***yiiT***	75.82	6.37/142	universal stress protein D
**RPS08 **	**Day 6 **	**CD03**	**Day 5**			***rbsB***	***priB,rbsP***	79.62	6.85/296	periplasmic ribose transperter protein
**RPS15 **	**Late-log (3 h) **					***rbsD***	***rbsP***	79.70	5.93/139	D-ribose pyranase; sugar-binding protein
**RPS29 **	**10 h **	**CD28**	**Day 3**			***yibJ***		83.35	5.00/?	RHA domain-containing protein YibJ
**RPS23 **	**Log **					***maoP***	***yifE***	85.06	6.09/112	macrodomain Ori protein
**RPS20 **	**Late-log (5 and 6 h) **					***hdeA***	***yhhC,yhiB***	85.74	5.06/110	periplasmic acid stress chaperone HdeA
**PRS12 **	**Late-log (3 h) **					***nfuA***	***gntY,yhbI***	88.12	4.52/191	iron-sulfur cluster carrier protein; gluconate transporter
**PRS14 **	**Late-log (3 h) **	**CD05**	**Day 3**			***slyD***		89.57	4.86/196	FKBP-type pepridyl-prolyl cis-trans isomerase
**PRS01 **	**Day 7 **					***X***				
**PRS13 **	**Late-log (3 h) **					***X***				
**PRS19 **	**Day 5 **					***X***				
**PRS27 **	**Late-log (2 h) **					***X***				
**PRS28 **	**Day 2 **					***X***				
**RPS30 **	**Day 7 **					***X***				
		**CD04 **	**Day 5**			***X***				
		**CD06**	**Late-log (3 h)**			***X***				
		**CD08**	**Log**			***X***				
		**CD10**	**Day 5 and 7**			***X***				
		**CD14**	**Day 6**			***X***				
		**CD16**	**Log**			***X***				
		**CD17**	**Log**			***X***				
		**CD18**	**Late-log (5 h)**			***X***				
		**CD20**	**Late-log (5 hr)**			***X***				
		**CD22**	**Day 8**			***X***				
		**CD26**	**Log**			***X***				

(green: PRS, orange: CD, and magenta: CR; see legends of **Figure 3** and **4**).

Furthermore, it is interesting to note that even in the last day 8, expressions of some stationary-phase proteins are synthesized, including HchA (protein/nucleic acid deglycase), Mdh (malate dehydrogenase), GuaB (inosine 5′-monophosphate dehydrogenase), and ZapB (cell division factor). HchA is involved in repair of glyoxal- and methylglyoxal-glycated proteins (Mihoub et al., 2015) and nucleic acids (Richarme et al., 2017). The *mdh* gene is also organized a network of genes, which facilitate stress-induced mutagenesis (Al Mamun et al., 2012). ZapB plays, together with ZapA, a role in organization and dynamics of the repaired genome in resting cells and independent of the Min system (Bailey et al., 2014; Mannik et al., 2016). Under stressful conditions unfavorable for *E. coli* growth, mutation rate increases for adaption and survival (Foster, 1999; Zinser and Kolter, 2004; Saint-Ruf et al., 2007). These 8-day proteins might be involved in repair of the genome and damaged proteins.

Both the sequential increase in cell buoyant density and the sequential synthesis of stationary-phase proteins are apparently under a single pathway, but it should be noted that the pathway for entry into the stationary phase is multiple. During the prolonged culture, the heterogeneity in the cell population should also be amplified due to generation of various types of cells on different pathways, such as persister cells, mutant cells, and dead cells (Roszak and Colwell, 1987; Kolter et al., 1993; Huisman et al., 1996; Ishihama, 1999).

## Growth Phase-Coupled Alterations in Gene Expression Apparatus

### Hibernation of the Transcription Apparatus

**Figure 5:**
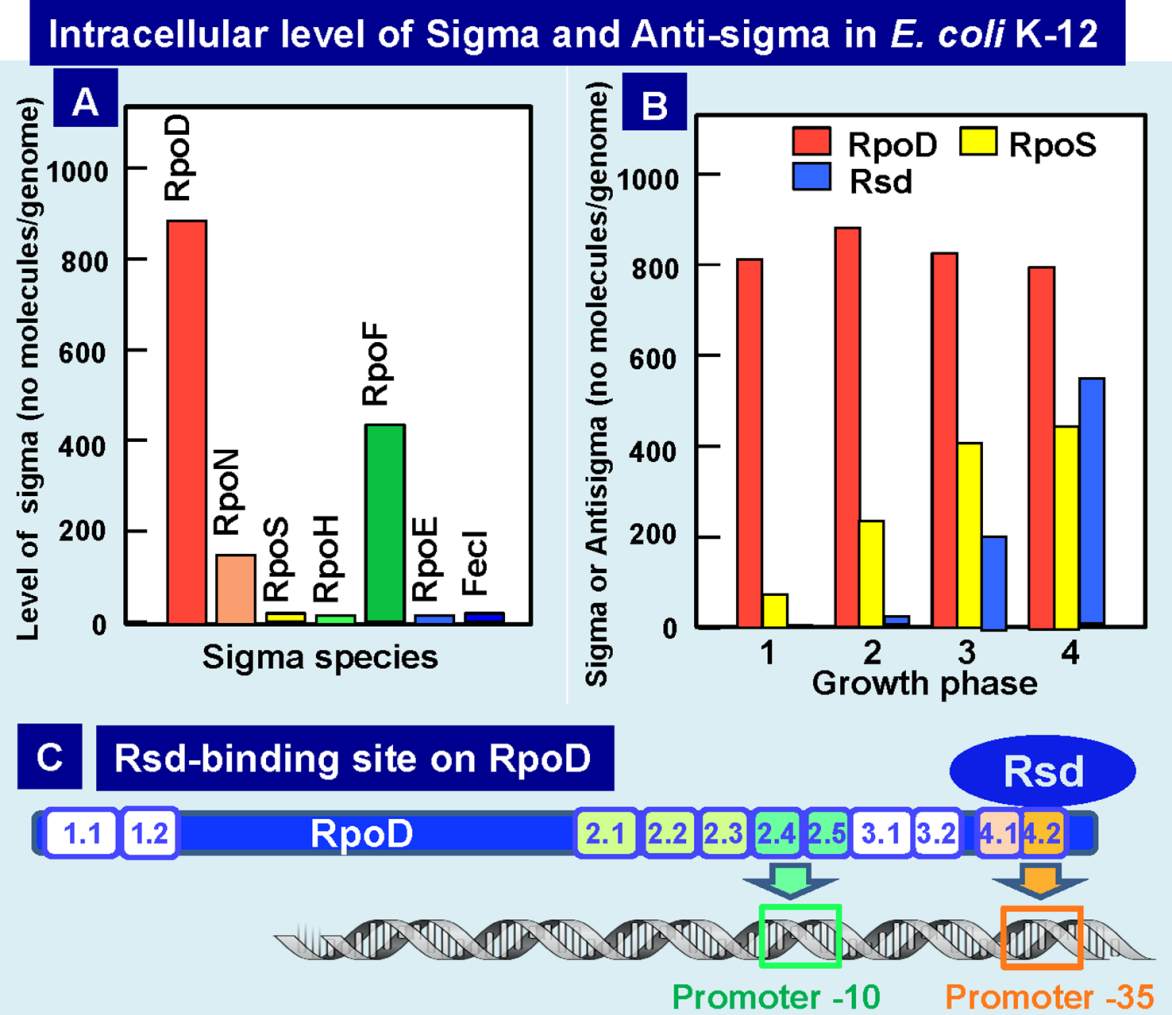
Intracellular levels of sigma factors and anti-RpoD sigma (Rsd). **(A)** Intracellular levels of all seven sigma factors in exponential phase *E. coli* K-12 was determined by Western blot analysis with use of specific antibodies (Jishage and Ishihama, 1995; Jishage et al., 1996). **(B)** Intracellular levels of growth-related RpoD sigma, stationary-phase-specific RpoS sigma, and anti-RpoD sigma Rsd were determined at various growth phases of *E. coli* K-12 (Jishage and Ishihama, 1998; Jishage and Ishihama, 1999). **(C)** The contact site of anti-sigma factor Rsd on the growth-related RpoD sigma was determined to be located within RpoD region-4 (promoter -35 recognition site) by using the contact-dependent cleavage sites by Rsd-tethered iron-*p*-bromoacetamidobenzyl EDTA by analysis of the complex formation between Ala-substituted σ^70^ and Rsd (Jishage and Ishihama, 2001). Rsd-binding to RpoD region-3 leads to silencing RpoD function.

Upon entry into the stationary phase, the level of transcription decreases to less than 10% of that in the log phase (Ishihama, 2000). For this marked reduction in transcription pattern, the modulation of the promoter selectivity of RNAP is the major mechanism through the replacement of sigma subunit (the promoter recognition factor). In *Escherichia coli* K-12, seven different species of the sigma subunit exist, each recognizing a specific set of promoters (Ishihama, 1988; Ishihama, 2010). Transcription of the genes highly expressed in exponential growth phase is carried out by the RNAP holoenzyme containing RpoD, while RpoS is a key factor in the change in genome expression during growth transition from the exponential growth phase to the stationary phase (Lowen and Hengge-Aronis, 1994; Ishihama, 2010; Ishihama, 2012). We have measured the intracellular level of each sigma subunit at various phases of cell growth (**Figure 5A**). In exponentially growing cells of *E. coli* K-12, a significant level was detected only for three sigma factors, RpoD for growth-related genes, RpoN for nitrogen-assimilation genes, and RpoF for flagella-chemotaxis genes (Ishihama et al., 1976; Kawakami et al., 1979; Jishage and Ishihama, 1995). The concentration of RpoD is maintained at a constant level of 500–700 molecules per genome from log to stationary phase. The log-phase cells contain 1,500 to 2,000 molecules of RNAP core enzyme per genome, but about two-third are involved in transcription cycle (Ishihama and Fukuda, 1980; Ishihama, 2000). After transcription initiation, RpoD sigma is released, and the majority of free RNAP core might be associated with RpoD sigma, forming the RpoD holoenzyme.

RpoS sigma is needed for transcription of stationary-phase genes. The level of RpoS starts to increase after the mid-log phase and reaches to the maximum level of about the half the level of RpoD in the stationary phase (**Figure 5B**) (Jishage and Ishihama, 1995; Jishage et al.,1996). The level of core enzyme is under the autogenous control, thereby keeping the constant level of about 2,000 molecules per genome throughout cell growth (Ishihama, 2000). In contrast, the combined level of all seven sigma factors is about two folds the level of the core enzyme, and we then proposed the “sigma competition” model (Jishage and Ishihama, 1998; Maeda et al., 2000). Since the level of RpoD was always higher than RpoS even after prolonged culture, we doubted whether RpoD is still functional in the stationary phase. As an attempt to examine this possibility, we analyzed proteins associated with RpoD at various phases of cell growth and discovered the association of a novel protein Rsd (regulator of sigma D) (Jishage and Ishihama, 1998; Jishage and Ishihama, 1999), which forms a complex with RpoD for interfering with its sigma function. The level of Rsd starts to increase upon entry into the stationary phase, finally reaching to the level of 60 to 80% of RpoD (**Figure 5B**), implying that most of RpoD stays non-functional in the stationary phase through formation of RpoD-Rsd complex. As a result, the core enzyme becomes available for association of the stationary-specific RpoS sigma (Jishage and Ishihama, 1998; Mitchell et al., 2007). The anti-sigma factor Rsd binds to the RpoD domain-4 that is involved in recognition of the promoter -35 signal (Dove and Hochschild, 2001; Jishage et al., 2001; Mitchell et al., 2007) (**Figure 5C**). Crystal structure of Rsd–RpoD complex supports this conclusion (Patikoglou et al., 2007). The affinity of Rsd to free RpoD is high, and in the presence of high concentrations of Rsd, it also binds to the core-associated RpoD (Ilag et al., 2004; Westblade et al., 2004). After sequestering RpoD into Rsd–RpoD complex, the free core enzyme could be used for formation of RpoS holoenzyme, thereby allowing transcription of stationary genes.

Besides RpoD sigma, Rsd was found to interact with HPr, a phosphocarrier component of PEP-dependent sugar-transporting phosphotransferase system (PTS), thereby interfering with anti-sigma activity (Park et al., 2013). Recently Rsd was also found to interact with SpoT and stimulates its hydrolysis activity of magic spot (p)ppGpp (Lee et al., 2018). The SpoT activity is, however, antagonized by dephosphorylated HPr, which generally interacts with a large number proteins and regulate wide varieties of carbon and energy metabolism (Rodionova et al., 2017). These observations altogether indicate the presence of a protein–protein interacting network between Rsd, HPr, and SpoT for interconnection between transcription and metabolism during the stationary phase.

Here, we propose the hibernation of growth-phase RNAP holoenzyme through conversion of RpoD sigma by Rsd anti-sigma factor. The RNAP core enzyme can then be used for assembly of RpoS holoenzyme for transcription of stationary-phase genes. It should be noted that excess free core enzyme, if present, should form transcriptionally inactive dimers or oligomers (Ishihama, 1990; Harris et al., 1995) for storage as in the case of yeast RNAP I (Fernandez-Tornero, 2018). The conversion of RpoD into the inactive RpoD-Rsd complex and the self-assembly of free core enzyme together contribute for silencing of the transcription apparatus during the stationary phase.

### Hibernation of the Translation Machinery

Bacterial ribosomes are universally conserved ribonucleoprotein complexes, generally consisting of two asymmetric subparticles. In *E. coli* K-21, large (50S) and small (30S) subparticles associate with each other to form the functional 70S ribosomes. The 50S subparticle is composed of two species of rRNA (23S and 5S) and a total of 33 species of the ribosomal protein, referred to r-protein (L1 to L36), whereas the 30S subparticle is composed of 16S rRNA and a total of 21 species of r-proteins (S1 to S21) (Wada and Sako, 1987; Izutsu et al., 2001; Kaczanowska and Ryden-Aulin, 2007; Shajani et al., 2011). Under optimal laboratory culture conditions, *E. coli* grows exponentially with heavy consumption of energy and resources.

**Figure 6:**
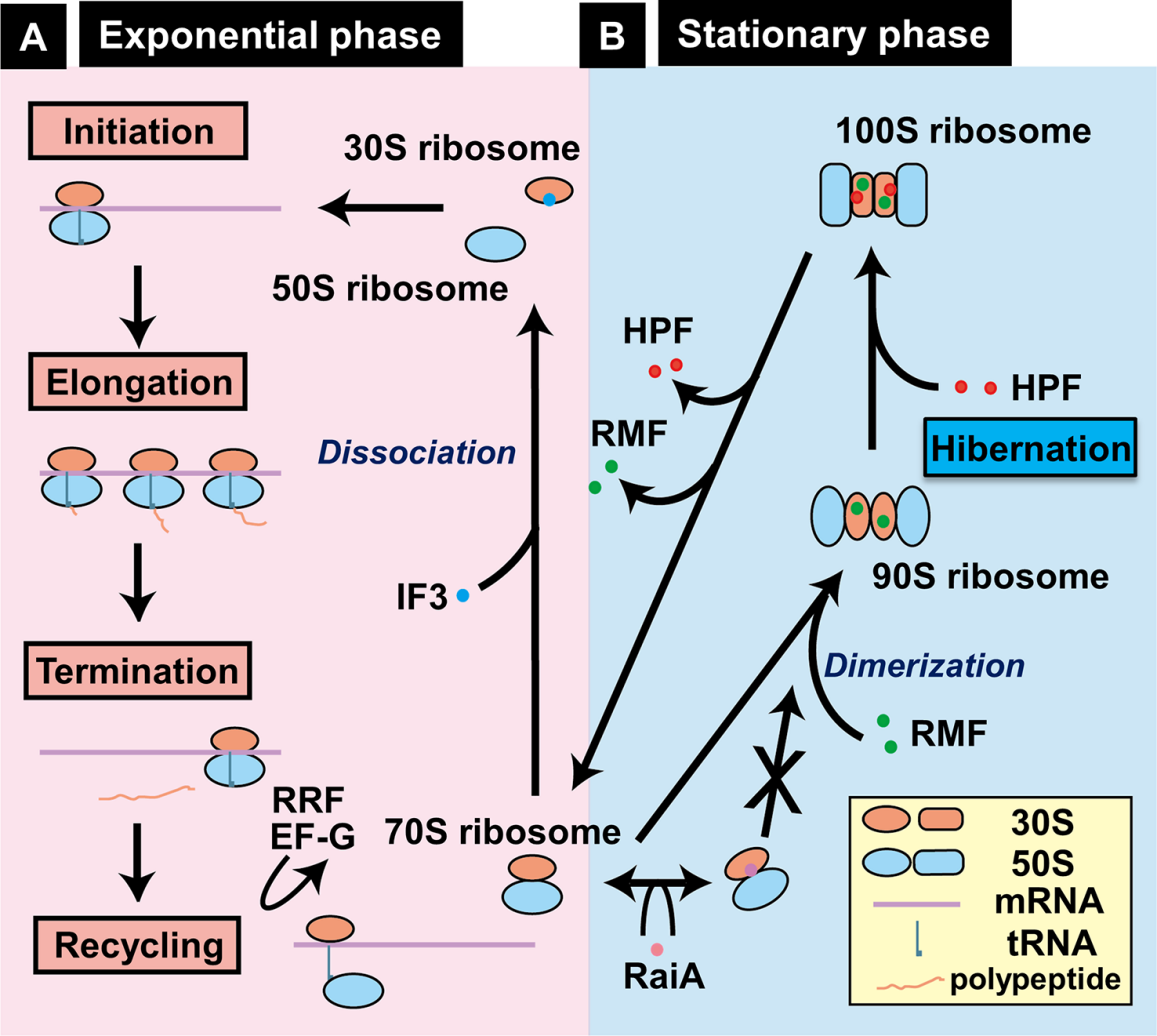
Growth phase-coupled alteration of ribosomes in *Escherichia coli* K-12. **(A)** In exponentially growing bacterial cells, most ribosomes are involved in the functional cycle of protein synthesis, consisting of initiation, elongation, termination, and recycling. For initiation, 30S and 50S ribosomes bind to mRNA, forming functional 70S ribosomes on mRNA and ultimately leading to form polysomes. After termination, 70S ribosomes are dissociated into 30S and 50S subparticles for reutilization. **(B)** Upon entry into stationary phase, unused ribosomes are converted into functionally inactive 100S dimeric ribosomes by sequential binding of RMF and HPF in *E. coli* K-12, one of Gram-negative bacteria (Wada, 1998; Yoshida and Wada, 2014). We designated this process as “hibernation.” Formation of 100S dimers is interfered by RaiA (renamed YfiA) (Ueta et al., 2005). The location of RMF on 30S ribosome is based on the recent cryo-electron micrography structure of 100S ribosome dimer (Beckert et al., 2018). By biochemical analyses, however, RMF was also indicated to bind 23S rRNA (Yoshida et al., 2004) and the peptidyl transferase center (Yoshida et al., 2002).

During this exponential phase, the ribosome profile detected by sucrose density gradient centrifugation (SDGC) includes 70S ribosomes as the major component and in addition, small amounts of 30S and 50S subparticles, and polysomes (**Supplemental Figure S3A**). These ribosomes are involved in the canonical ribosome cycle (initiation, elongation, termination, and recycling) of protein synthesis (**Figure 6A**). Protein synthesis is the most energy demanding cellular process. The majority of metabolic energy is used for the formation of ribosomes (Maaloe and Kjeldgaard, 1966). Upon entry into the stationary phase, overall level of transcription decreases to less than 10% the level of log phase, yielding the superfluous translation machinery. The unused excess ribosomes are then converted into non-functional 100S ribosome dimers, the inactive stored form of ribosomes (**Supplemental Figure S3B** and **Figure 6B**) (Wada et al., 1990; Yoshida and Wada, 2014). The ribosome profile measured by SDGC includes a peak of 100S ribosomes besides the peak of 30S, 50S, and 70S ribosome (**Supplemental Figure S3B** and **Figure 6B**). The 100S ribosome is a dimer of 70S ribosomes, and inactive in translation (Wada et al., 1990; Wada et al., 1995). We then designated this stage of ribosome cycle, in which the ribosomes stay in inactive forms, for “Hibernation” (Yoshida et al., 2002).

The 100S ribosome of *E. coli* is formed by the binding of two factors, the RMF (Wada et al., 1990) and the HPF (Ueta et al., 2013). RMF alone leads only to the formation of 90S particle, which is an immature form of the 100S ribosome, suggesting that HPF is needed to convert this premature 90S particle to mature 100S ribosome (Ueta et al., 2005; Ueta et al., 2008; Ueta et al., 2013). The third protein associated with the stationary-phase ribosomes is RaiA (renamed YfiA), which interferes with the 100S dimer formation through competition with HPF binding (Maki et al., 2000; Ueta et al., 2005). Thus, two factors, HPF and RaiA, share the same binding site on the 100S ribosome and thus compete each other, thereby controlling the formation of 100S ribosomes. The binding sites of RMF and HPF investigated by several methods indicate the conformational changes of 30S subunits, thereby controlling the ribosome dimerization indirectly (Yoshida et al., 2002; Ueta et al., 2005; Yoshida and Wada, 2014; Beckert et al., 2018) (see **Figure 6**, right panel). Inactivation of the *rmf* gene leads to loss of viability in the stationary phase (Yamagishi et al., 1993), under acidic conditions (El-Sharoud and Niven, 2007) and upon exposure to heat shock (Niven, 2004). When the stationary-phase *E. coli* was transferred to nutrient-rich media, the disassembly of 100S ribosomes is rapid within 1 min (Aiso et al., 2005) for restart of protein synthesis (Yoshida and Wada, 2014). The mechanism how RMF and HPF are removed from 100S ribosomes remains to be solved.

The ribosome hibernation is widespread but the factors involved in this process are different between bacteria (Ueta et al., 2008; Yoshida and Wada, 2014; Prossliner et al., 2018). *E. coli* and some γ-proteobacteria carry both the *rmf* and *hpf* genes, but many other bacteria have only the *hpf* gene or its homologue devoid of the *rmf* gene (Ueta et al., 2008). In bacteria carrying a long-type HPF homologue, the ribosome dimerization takes place in the absence of RMF (Ueta et al., 2013; Akanuma et al., 2016). *E. coli* forms 100S ribosomes only in their stationary growth phase, but in Gram-positive bacteria such as *Bacillus subtilis*, 100S ribosomal dimers are formed throughout entire growth phases (Ueta et al., 2013; Puri et al., 2014; Akanuma et al., 2016), implying that the factors or conditions for ribosome dimerization are different between bacterial species.

In the case of the bacterial group having long HPF, several structures have been proposed for the ribosome dimer (For instance, Matzov et al., 2019). Accordingly, the 70S–70S interface within ribosome dimers appeared different from that of *E. coli* (Kato et al., 2010; Beckert et al., 2018). Nevertheless, N-terminal domain of long HPF is predicted to bind to the site overlapping with the tRNA-binding site as in the case of HPF in *E. coli*, suggesting that common mechanism of translational silencing exists between bacteria carrying long and short HPFs.

## Coordinated Hibernation of Transcription Apparatus and Translation Machinery

The formation of transcriptional apparatus and translational machinery are tightly coupled and coordinated, showing the growth rate-dependent synthesis of RNAP core enzyme (Ishihama and Fukuda, 1980; Ishihama, 1988) and ribosomes (Nomura et al., 1984; Zengel and Lindahl, 1994), thereby keeping the ratio of 5∼10 ribosomes per RNAP core to match effective translation of mRNA through formation of polysomes. For this purpose, multiple layers of regulation are involved such as the organization of genes for RNAP subunits and ribosomal proteins into single and same operons, and the autogenous regulation of synthesis of RNAP subunits and ribosomal proteins by excess and unused products. We then examined the possible coordination in the hibernation process between transcription apparatus and translation machinery. During the growth transition of *Escherichia coli* from log to stationary phase, the level of genome expression is reduced less than 10% the log-phase level and the pattern of genome expression (the species of expressed genes) is also markedly modulated. For this alteration, the transcription apparatus is altered by binding of anti-sigma factor Rsd to the RpoD sigma for sigma replacement with stationary-phase-specific RpoS (see above) while the translation machinery is modulated by binding of RMF and HPF to 70S ribosome to form the inactive 100S ribosome dimer (see above). Until recently, however, little was known how the expression of factors involved in hibernation of transcription apparatus and translation machinery is regulated. We have then performed a systematic search for TFs involved in regulation of the promoters of two key regulators, Rsd for hibernation of RNAP and RMF for hibernation of ribosomes, by using the newly developed promoter-specific transcription factor (PS-TF) screening system (Shimada et al., 2013; Yoshida et al., 2018).

**Figure 7:**
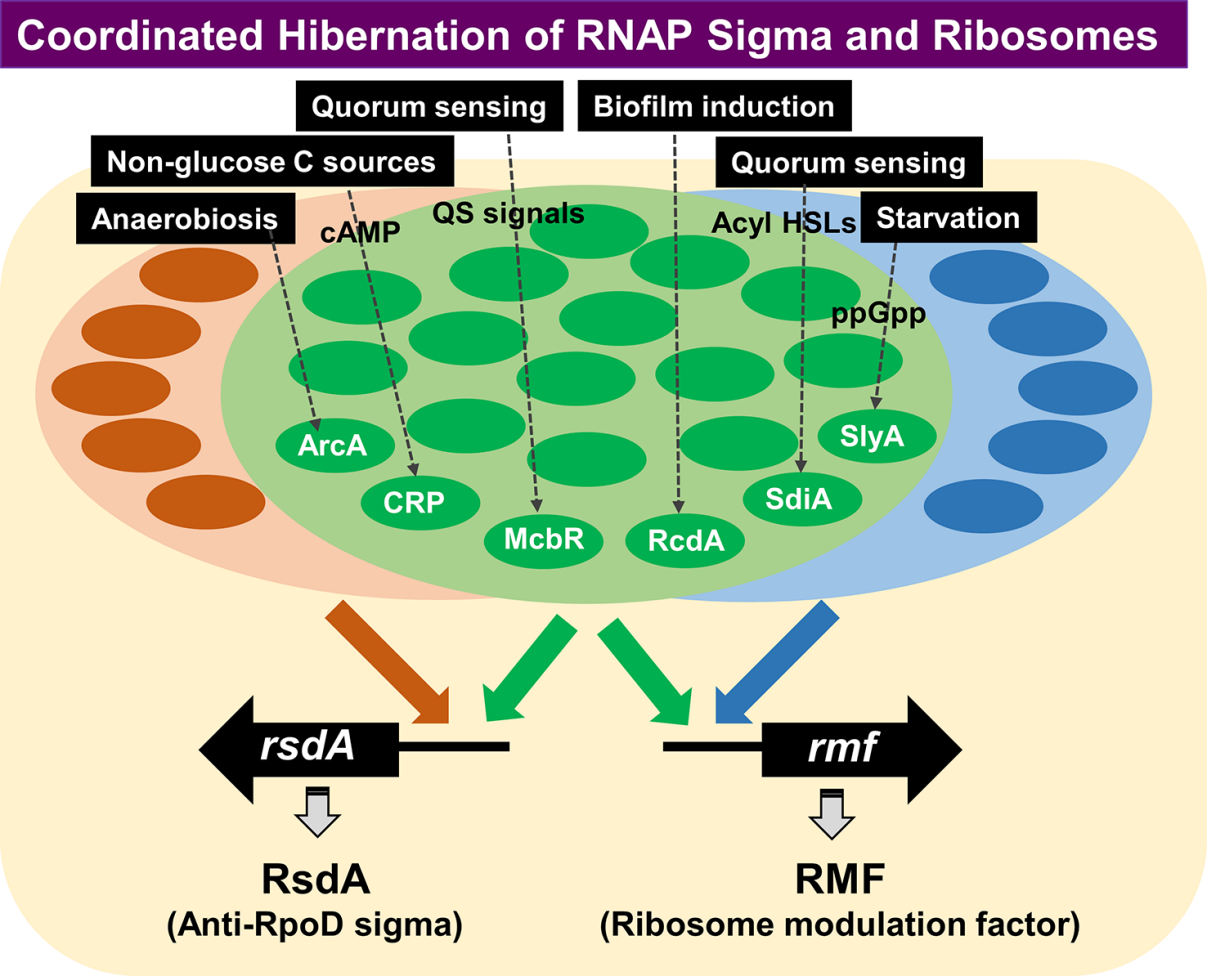
PS-TF screening was performed for search of TFs involved in regulation of the *rsd* and *rmf* genes. A total of 74 TF species were found to bind to both the *rsd* and *rmf* promoter probes, although the binding affinity appeared different between these TFs (Yoshida et al., 2018). Besides these 74 TFs, some other TFs have been identified to bind only the *rsd* gene or the *rmf* gene, indicating independent regulation of the two genes under as yet unidentified conditions. Detailed analysis of the regulatory roles *in vitro* and *in vivo* was performed for the five representative stress-response TFs (ArcA, McbR, RcdA, SdiA, and SlyA) (Yoshida et al., 2018). ArcA was indicated to repress transcription of both *rsd* and *rmf* genes, while other four were suggested to activate both genes. gSELEX indicated that all these TFs regulate not only the *rsd* and *rmf* genes but also regulate a number of genes supposedly required for survival under stressful conditions. PS-TF, promoter-specific transcription factor.

Using *rsd* and *rmf* promoter probes and a total of about 200 purified TFs from *E. coli* K-12 W3110, we performed PS-TF screening (Yoshida et al., 2018). A total of 74 TF species (55 group A TFs and 19 group B TFs) were found to bind to both the *rsd* and *rmf* probes, although the binding affinity was different between these TFs (Yoshida et al., 2018), suggesting that both the *rmf* and *rsd* genes are under the control of multi-factor promoters (Ishihama et al., 2016). After repetition of PS-TF, we succeeded to focus on a total of 19 TFs, of which 9 (ArcA, CRP, CueR, McbR, NhaR, RcdA, SdiA, SlyA, and ZntR) have been experimentally confirmed to be involved in regulation *in vitro* and *in vivo* of both the *rsd* and *rmf* genes (Yoshida et al., 2018) (**Figure 7**). The synthesis of RMF is also under the control of ppGpp (Izutsu et al., 2001). Results altogether indicated the involvement of a common set of TFs, each sensing a specific but different environmental condition, in coordinated hibernation of the transcriptional apparatus and translational machinery for adaptation and survival under stressful conditions. Translation of RMF is stimulated by polyamines (Terui et al., 2010), which accumulates in the stationary phase (Igarashi and Kashiwagi, 2018).

Besides the large set of TFs with binding activity to both *rsd* and *rmf* probes, a small number of TFs bound only to either the *rsd* or *rmf* probe (**Figure 7**). This finding indicates the two key players for hibernation of transcription apparatus and translational machinery are regulated independently under certain specific conditions. These *rsd*- or *rmf-*specific TFs might be involved in independent regulation of either transcriptional apparatus or translational machinery under as yet unidentified specific environmental conditions. This review proves the initial stage of molecular basis of the hibernation of *E. coli*, focusing on the transcription apparatus and the translation machinery. The whole set of TFs involved in the regulation of *rsd* and *rmf* genes will be described elsewhere.

## Author Contributions

HY: data collection, writing, and figure preparation in Hibernation of the Translation Machinery and Coordinated Hibernation of Transcription Apparatus and Translation Machinery sections. TS: data collection and figure preparation in Discontinuous Change of the Cell Buoyant Density and Coordinated Hibernation of Transcription Apparatus and Translation Machinery. AW and YM: data collection, writing, and figure preparation in Growth-Dependent Change of the Protein Expression Pattern section. AI: design of this review article, data collection, writing, and figure preparation in Introduction, Discontinuous Change of the Cell Buoyant Density, Hibernation of the Transcription Apparatus, and Coordinated Hibernation of Transcription Apparatus and Translation Machinery. All authors read and approved the manuscript.

## Funding

This study was supported by MEXT Cooperative Research Program of Network Joint Research Center for Materials and Devices to AI, and the MEXT-Supported Program for the Strategic Research Foundation at Private Universities to AI.

## Conflict of Interest

The authors declare that the research was conducted in the absence of any commercial or financial relationships that could be construed as a potential conflict of interest.

## Supplementary Material

Supplemental Figure S1 RFHR 2D gel patterns of *E. coli* proteins. *E. coli* K-12 AD202 was grown at 37 °C in minimal medium E containing 2% peptone. Cell lysates were prepared as described in text, and fractionated by centrifugation into CD (Insoluble cell debris), CE (supernatant cell extract), CR (crude ribosome), and PRS (post ribosomal supernatant) fractions. Soluble proteins of the CR [A], PRS [B], basic CD [C] and acidic CD [C] were subjected to RFHR (radical free and highly reducing) method of 2D gel analysis (Wada 1986a; Wada, 1986b). Gels were stained with CBB.

Supplemental Figure S2 Contents of some growth-related proteins in cells fractionated by Percoll centrifugation. *E. coli* K-12 was grown at 37°C in LB medium for 12 hrs. Cell suspension was directly subjected to Percoll gradient centrifugation under the standard procedure (Makinoshima et al., 2002; Makinoshima et al., 2003). The gradient was fractionated and the content of some representative growth-related proteins in each fraction was determined by Western blot analysis with use of specific antibodies (Ishihama et al., 2014).

Supplemental Figure S3 Ribosomes were subjected to sucrose density gradient centrifugation (SDGC) (Wada et al., 1990). **(A)** The pattern of ribosomes from exponential phase includes 70S ribosome as the major component and in addition, small amounts of 30S and 50S subparticles, and polysomes. **(B)** Ribosomes from stationary phase showed 100S dimeric ribosomes in addition to 30S, 50S and 70S ribosomes.

Supplemental Table S1 Proteins Expressed During Prolonged Culture of Escherichia coli K-12 PRS (Post Ribosomal Supernatant) fraction

## References

[B1] Aiso T. Yoshida H. Wada A. Ohki R. (2005). Modulation of mRNA stability participates in stationary-phase specific expression of ribosome modulation factor. J. Bacteriol. 187, 1951–1958. 10.1128/JB.187.6.1951-1958.2005 15743942 PMC1064039

[B2] Akanuma G. Kazo Y. Tagami K. Hiraoka H. Yano K. Suzuki S. (2016). Ribosome dimerization is essential for the efficient regrowth of Bacillus subtilis. Microbiology 162, 448–458. 10.1099/mic.0.000234 26743942

[B3] Al Mamun A. A. M. Lombardo M.-J. Shee C. Lisewski A. M. Gonzalez C. Lin D. (2012). Identity and function of a large gene network underlying mutagenic repair of DNA breaks. Science 338, 1344–1348. 10.1126/science.1226683 23224554 PMC3782309

[B4] Baba T. Ara T. Hasegawa M. Takai Y. Okumura Y. Baba M. (2006). Construction of *Escherichia coli* K-12 in-frame, single-gene knockout mutants: the Keio collection. Mol. Syst. Biol. 2, 20060008. 10.1038/msb4100050 PMC1681482 16738554

[B5] Bailey M. W. Bisicchia P. Warren B. T. Sherratt D. J. Mannik J. (2014). Evidence for divisome localization mechanisms independent of the Min system and SlmA in *Escherichia coli* . PloS Genet. 10, e1004504. 10.1371/journal.pgen.1004504 25101671 PMC4125044

[B6] Battesti A. Majdaiani N. Gottesman S. (2011). The RpoS-mediated general stress response in *Escherichia coli* . Annu. Rev. Microbiol. 65, 189–213. 10.1146/annurev-micro-090110-102946 21639793 PMC7356644

[B7] Beckert B. Turk M. Czech A. Berninghausen O. Beckmann R. Ignatova Z. (2018). Structure of a hibernating 100S ribosome reveals an inactive conformation of the ribosomal protein S1 . Nat. Microbiol. 3, 1115–1121. 10.1038/s41564-018-0237-0 30177741

[B8] Caglar M. U. Hockenberry A. J. Wilke C. O. (2018). Predicting bacterial growth conditions from mRNA and protein abundances. PloS One 13, e0206634. 10.1371/journal.pone.0206634 30388153 PMC6214550

[B9] Dove S. L. Hochschild A. (2001). Bacterial two-hybrid analysis of interactions between region 4 of the σ70 subunit of RNA polymerase and the transcription regulators Rsd from *Escherichia coli* and AlgQ from *Pseudomonas aeruginosa* . J. Bacteriol. 183, 6413–6421. 10.1128/JB.183.21.6413-6421.2001 11591686 PMC100137

[B10] Dwek R. D. Kobrin L. H. Grossman N. Ron E. Z. (1980). Synchronization of cell division in microorganisms by Percoll gradients. J. Bacteriol. 144, 17–21. 6252189 10.1128/jb.144.1.17-21.1980 PMC294577

[B11] El-Sharoud W. M. Niven G. W. (2007). The influence of ribosome modulation factor on the survival of stationary-phase *Escherichia coli* during acid stress. Microbiology 153, 247–253. 10.1099/mic.0.2006/001552-0 17185553

[B12] Ferenci T. (2001). Hungry bacteria – definition and properties of a nutritional state. Environ. Microbiol. 3, 605–611. 10.1046/j.1462-2920.00238.x 11722540

[B13] Fernandez-Tornero C. (2018). RNA polymerase I activation and hibernation: unique mechanisms for unique genes. Transcription 9, 248–254. 10.1080/21541264.2017.1416267 29372670 PMC6104688

[B14] Foster P. L. (1999). Mechanisms of stationary phase mutation: a decade of adaptive mutation. Annu. Rev. Genet. 33, 57–88. 10.1146/annurev.genet.33.1.57 10690404 PMC2922758

[B15] Franchini A. G. Ihssen J. Egli T. (2015). Effect of global regulators RpoS and cyclic-AMP/CRP on the catabolome and transcriptome of *Escherichia coli* K12 during carbon- and energy-limited growth. PloS One 10, e0133793. 10.1371/journal.pone.0133793 eCollection 2015. 26204448 PMC4512719

[B16] Harris S. J. Williams R. C. Jr. Lee J. C. (1995). Self-association of *Escherichia coli* DNA-dependent RNA polymerase core enzyme. Biochemistry 34, 8752–8762. 10.1021/bi00027a026 7612615

[B17] Helmann J. D. (1999). Anti-sigma factors. Curr. Opin. Microbiol. 2, 135–141. 10.1016/S1369-5274(99)80024-1 10322161

[B18] Hofmann N. Wurm R. Wagner R. (2011). The *E. coli* anti-sigma factor Rsd: Studies on the specificity and regulation of its expression. PloS One 6, e19235. 10.1371/journal.pone.0019235 21573101 PMC3089606

[B19] Hughes K. T. Mathee K. (1998). The anti-sigma factors. Annu. Rev. Microbiol. 52, 231–286. 10.1146/annurev.micro.52.1.231 9891799

[B20] Huisman G. W. Siegele D. A. Zambrano M. M. Kolter R. (1996). Morphological and physiological changes during stationary phase, in Escherichia coli and Salmonella. Ed. Neidhardt F. C. (Washington, DC: American Society for Microbiology Press), 1672–1682.

[B21] Igarashi K. Kashiwagi K. (2018). Effect of polyamine on protein synthesis and growth. J. Biol. Chem. 293, 18702–18707. 10.1074/jbc.TM118.003465 30108177 PMC6290148

[B22] Ilag L. L. Westblade L. F. Deshayes C. Kolb A. Busby S. J. W. Robinson C. V. (2004). Mass spectrometry of *Escherichia coli* RNA polymerase: Interactions of the core enzyme wtih σ^70^ and Rsd protein. Structure 12, 269–275. 10.1016/j.str.2004.01.007 14962387

[B23] Ishihama A. Taketo M. Saitoh T. Fukuda R. (1976). Control of formation of RNA polymerase in Escherichia coli, in RNA Polymerase. Ed. Chamberlin M. Losick R. (New York, Cold Spring Harbor Laboratory Press), 485–502.

[B24] Ishihama A. Fukuda R. (1980). Autogenous and post-transcriptional regulation of RNA polymerase synthesis. Mol. Cell. Biochem. 31, 177–196. 10.1007/bf00225850 7003354

[B25] Ishihama A. Shimada T. Yamazaki Y. (2016). Transcription profile of *Escherichia coli:* genomic SELEX search for regulatory targets of transcription factors. Nucleic Acids Res. 44, 2058–2074. 10.1093/nar/gkw051 26843427 PMC4797297

[B26] Ishihama A. (1988). Promoter selectivity of prokaryotic RNA polymerase. Trends Genet. 4, 282–286. 10.1016/0168-9525(88)90170-9 3076288

[B27] Ishihama A. (1990). Molecular assembly and functional modulation of *Escherichia coli* RNA polymerase. Adv. Biophys. 26, 19–31. 10.1016/0065-227x(90)90005-e 2082727

[B28] Ishihama A. (1997). Adaptation of gene expression in stationary phase bacteria. Curr. Opin. Genet. Dev. 7, 582–588. 10.1016/S0959-437X(97)80003-2 9388772

[B29] Ishihama A. (1999). Modulation of the nucleoid, the transcription apparatus, and the translation machinery in bacteria for stationary phase survival. Genes Cells 3, 135–143. 10.1046/j.1365-2443.1999.00247.x 10320479

[B30] Ishihama A. (2000). Functional modulation of *Escherichia coli* RNA polymerase. Annu. Rev. Microbiol. 54, 499–518. 10.1146/annurev.micro.54.1.499 11018136

[B31] Ishihama A. (2009).The nucleoid: an overview. EcoSal—Escherichia coli and Salmonella: Cellular and Molecular Biology. Neidhardt FC Washington, DC: American Society for Microbiology Press, 1672–1682.

[B32] Ishihama A. (2010). Prokaryotic genome regulation: multi-factor promoters, multi-target regulators and hierarchic networks. FEMS Microbial. Rev. 34, 628–645. 10.1111/j.1574-6976.2010.00227.x 20491932

[B33] Ishihama A. (2012). Prokaryotic genome regulation: a revolutionary paradigm. Proc. Jpn. Acad. Ser. B Phys. Biol. Sci. 88, 485–508. 10.2183/pjab.88.485 PMC3511978 23138451

[B34] Ishihama A. Kori A. Koshio E. Yamada K. Maeda H. Shimada T. (2014). Intracellular concentrations of 65 species of transcription factors with known regulatory functions in *Escherichia coli* . J. Bacteriol. 196, 2718–2727. 10.1128/JB.01579-14 24837290 PMC4135669

[B35] Izutsu K. Wada C. Komine Y. Sako T. Ueguchi C. Nakura S. (2001). *Escherichia coli* ribosome-associated protein SRA, whose copy number increases during stationary phase. J. Bacteriol. 183, 2765–2773. 10.1046/j.1365-2443.2001.00457.x 11292794 PMC99491

[B36] Jaworski A. Higgins N. P. Wells R. D. Zacarias W. (1991). Topoisomerase mutants and physiological conditions control supercoiling and Z-DNA formation *in vivo* . J. Biol. Chem. 266, 2576–2581. 1846630

[B37] Jin D. J. Cagliero C. Zhou Y. N. (2012). Growth rate regulation in *Escherichia coli* . FEMS Microbiol. Rev. 36, 269–287. 10.1111/j.1574-6976.2011.00279.x 21569058 PMC3478676

[B38] Jishage M. Ishihama A. (1995). Regulation of RNA polymerase sigma subunit synthesis in *Escherichia coli*: intracellular levels of σ^70^ and σ^38^ . J. Bacteriol. 177, 6832–6835. 10.1128/jb.178.18.5447-5451.1996 7592475 PMC177550

[B39] Jishage M. Ishihama A. (1998). A stationary phase protein in *Escherichia coli* with binding activity to the major sigma subunit of RNA polymerase. Proc. Natl. Acad. Sci. U.S.A. 95, 4953–4958. 10.1073/pnas.95.9.4953 9560209 PMC20194

[B40] Jishage M. Ishihama A. (1999). Transcriptional organization and *in vivo* role of the *Escherichia coli rsd* gene, encoding the regulator of RNA polymerase sigma D. J. Bacteriol. 181, 3768–3776. 10368152 10.1128/jb.181.12.3768-3776.1999 PMC93855

[B41] Jishage M. Iwata A. Ueda S. Ishihama A. (1996). Regulation of RNA polymerase sigma subunit synthesis in *Escherichia coli*: Intracellular levels of four species of sigma subunit under various growth conditions. J. Bacteriol. 178, 5447–5451. 10.1128/jb.178.18.5447-5451.1996 8808934 PMC178365

[B42] Jishage M. Dasgupta D. Ishihama A. (2001). Mapping of the Rsd contact site on the sigma-70 subunit of *Escherichia coli* RNA polymerase. J. Bacteriol. 183, 2952–2956. 10.1128/JB.183.9.2952-2956.2001 11292818 PMC99515

[B43] Kaczanowska M. Ryden-Aulin M. (2007). Ribosome biogenesis and the translation process in *Escherichia coli* . Microbiol. Mol. Biol. Rev. 71, 477–494. 10.1128/MMBR.00013-07 17804668 PMC2168646

[B44] Kato T. Yoshida H. Miyata T. Maki Y. Wada A. Namba K. (2010). Structure of the 100S ribosome in the hibernation stage revealed by electron cryomicroscopy. Structure 18, 719–724. 10.1016/j.str.2010.02.017 20541509

[B45] Kawakami K. Saitoh T. Ishihama A. (1979). Biosynthesis of RNA polymerase in *Escherichia coli.* IX. Growth-dependent variations in the synthesis rate, content and distribution of RNA polymerase. Mol. Gen. Genet. 174, 107–116. 10.1007/bf00268348 386039

[B46] Koch A. L. (1996). Similarities and differences of individual bacteria within a clone, in Escherichia coli and Salmonella. Ed. Neidhardt F. C. (Washington, DC: American Society for Microbiology Press), 1640–1661.

[B47] Kolter R. Siegele D. A. Tormo A. (1993). The stationary phase of the bacterial life cycle. Annu. Rev. Microbiol. 47, 855–874. 10.1146/annurev.mi.47.100193.004231 8257118

[B48] Kubitschek H. E. Baldwin W. W. Graetzer R. (1983). Buoyant density constancy during the cell cycle of *Escherichia coli* . J. Bacteriol. 155, 1027–1032. 6350259 10.1128/jb.155.3.1027-1032.1983 PMC217795

[B49] Kusano S. Ding Q. Fujita N. Ishihama A. (1996). Promoter selectivity of *Escherichia coli* RNA polymerase Eσ^70^ and Eσ^38^ holoenzymes: effect of DNA supercoiling. J. Biol. Chem. 271, 1998–2004. 10.1074/jbc.271.4.1998 8567650

[B50] Lee J.-W. Park Y.-H. Seok Y.-J. (2018). Rsd balances (p)ppGpp level by stimulating the hydrolase activity of SpoT during carbon source downshift in. Escherichia coli. Proc. Natl. Acad. Sci. U.S.A. 115, E6845–E6854. 10.1073/pnas.1722514115 29915072 PMC6055147

[B51] Lowen P. C. Hengge-Aronis R. (1994). The role of the sigma factor σ^s^ (*katF*) in bacterial global regulation. Annu. Rev. Microbiol. 48, 53–80. 10.1146/annurev.mi.48.100194.000413 7826018

[B52] Maaloe O. Kjeldgaard N. O. (1966).Control of macromolecular synthesis. Inc., New York: W.A. Benjamin.

[B53] Maeda H. Fujita N. Ishihama A. (2000). Competition among seven *Escherichia coli* sigma subunits: relative binding affinities to the core RNA polymerase. Nucleic Acids Res. 28, 3497–3503. 10.1093/nar/28.18.3497 10982868 PMC110723

[B54] Maki Y. Yoshida H. Wada A. (2000). Two proteins, YfiA and YhbH, associated with resting ribosomes in stationary phase *Escherichia coli* . Genes Cells 5, 965–974. 10.1046/j.1365-2443.2000.00389.x 11168583

[B55] Makinoshima H. Nishimura A. Ishihama A. (2002). Fractionation of *Escherichia coli* cell populations at different stages during growth transition to stationary phase. Mol. Microbiol. 43, 269–279. 10.1046/j.1365-2958.2002.02746.x 11985708

[B56] Makinoshima H. Aizawa S. Hayashi H. Miki T. Nishimura A. Ishihama A. (2003). Growth-phase-coupled alterations in cell structure and function of *Escherichia coli* . J. Bacteriol. 185, 1338–1345. 10.1128/jb.185.4.1338-1345.2003 12562804 PMC142870

[B57] Mannik J. Castillo D. E. Yang D. Siopsis G. Mannik J. (2016). The role of MatP, ZapA and ZapB in chromosomal organization and dynamics in *Escherichia coli* . Nucleic Acids Res. 44, 1216–1226. 10.1093/nar/gkv1484 26762981 PMC4756834

[B58] Martinez-Antonio A. Lomnitz J. G. Sandoval S. Aldana M. Savgeau M. A. (2012). Regulatory design governing progression of population growth phases in bacteria. PloS One 7, e30654. 10.1073/pnas.1722514115 22363461 PMC3283595

[B59] Matzov D. Bashan A. Yap M. F. Amunts A. Yonath A. (2019). Stress response as implemented by hibernating ribosomes: a structural overview. FEBS J. 286, 3558–3565. 10.1111/febs.14968 31230411 PMC6746590

[B60] Mehta P. Jovanovic G. Ying L. Buck M. (2015). Is the cellular and molecular machinvery in the stationary phase of *Escherichia coli*?. Biochem. Soc Trans. 43, 168–171. 10.1042/BST20140267 25849912

[B61] Mihoub M. Abdallah J. Gontero B. Dairou J. Richarme G. (2015). The DJ-1 superfamily member Hsp31 repairs proteins from glycation by methylglyoxal and glyoxal. Biochem. Biophys. Res. Commun. 463, 1305–1310. 10.1016/j.bbrc.2015.06.111 26102038

[B62] Mitchell J. E. Oshima T. Piper S. E. Webster C. L. Westblade L. F. Karimova G. (2007). The *Escherichia coli* regulator of σ^70^ protein, Rsd, can up-regulate some stress-dependent promoters by sequestering σ^70^ . J. Bacteriol. 189, 3489–3495. 10.1128/JB.00019-07 17351046 PMC1855875

[B63] Nachin L. Nanmark U. Nystrom T. (2005). Differential roles of the universal stress proteins of *Escherichia coli* in oxidative stress resistance, adhesion, and motility. J. Bacteriol. 187, 6265–6272. 10.1128/JB.187.18.6265-6272.2005 16159758 PMC1236625

[B64] Niven G. W. (2004). Ribosome modulation factor protects *Escherichia coli* during heat stress, but this may not be dependent on ribosome dimerization. Arch. Microbiol. 182, 60–66. 10.1007/s00203-004-0698-9 15278243

[B65] Nomura M. Gource R. Baghman G. (1984). Regulation of the synthesis of ribosomes and ribosomal components. Annu. Rev. Biochem. 53, 75–117. 10.1146/annurev.bi.53.070184.000451 6206783

[B66] Paget M. S. (2015). Bacterial sigma factors and anti-sigma factors: Structure, function and distribution. Biomolecules 5, 1245–1265. 10.3390/biom5031245 26131973 PMC4598750

[B67] Park Y. H. Lee C. R. Choe M. Seok Y. J. (2013). HPr antagonizes the anti-s70 activity of Rsd in *Escherichia coli* . Proc. Natl. Acad. Sci. U.S.A. 110, 21142–21147. 10.1073/pnas.1316629111 24324139 PMC3876255

[B68] Patikoglou G. A. Westblade L. F. Campbell E. A. Lanour V. Lane W. J. Darst S. A. (2007). Crystal structure of the *Escherichia coli* regulator of σ^70^, Rsd, in complex with σ^70^ domain 4 . J. Mol. Biol. 21, 649–659. 10.1016/j.jmb.2007.06.081 PMC2083641 17681541

[B69] Pletnev P. Osterman I. Sergiev P. Bogdanov O. Dontsova O. (2015). Survival guide: *Escherichia coli *in the stationary phase. Acta Nat. 7, 22–33. PMC4717247 26798489

[B70] Prossliner T. Skovbo Winther K. Serensen M. A. Gerdes K. (2018). Ribosome hibernation. Ann. Rev. Genet. 52, 321–348. 10.1146/annurev-genet-120215-035130 30476446

[B71] Puri P. Eckhardt T. H. Franken L. E. Fusetti F. Stuart M. C. Boekema E. J. (2014). Lactococcus lactis YfiA is necessary and sufficient for ribosome dimerization. Mol. Microbiol. 91, 394–407. 10.1111/mmi.12468 24279750

[B72] Raivio T. L. (2005). Envelope stress responses and Gram-negative bacterial pathogenesis. Mol. Microb. 56, 1119–1128. 10.1111/j.1365-2958.2005.04625.x 15882407

[B73] Richarme G. Liu C. Mihoub M. Abdallah J. Leger T. Joly N. (2017). Gualine glycation repair by DJ-1/Park7 and its bacterial homologs. Science 357, 208–211. 10.1126/science.aag1095 28596309

[B74] Rodionova I. A. Zhang Z. Mehla J. Goodacre N. Babum N. Emili A. (2017). The phosphocarrier protein HPr of the bacterial phosphotransferase system globally regulates energy metabolism by directly interacting with multiple enzymes in*Escherichia coli* . J. Biol. Chem. 292, 14250–14257. 10.1074/jbc.M117.795294 28634232 PMC5572926

[B75] Roszak D. B. Colwell R. R. (1987). Survival strategies of bacteria in the natural environment. Microbiol. Rev. 51, 365–379. 3312987 10.1128/mr.51.3.365-379.1987 PMC373117

[B76] Saint-Ruf C. Pesut J. Sopta M. Matic I. (2007). Causes and consequences of DNA repair activity modulation during stationary phase in *Escherichia coli* . Crit. Rev. Biochem. Mol. Biol. 42, 259–270. 10.1080/10409230701495599 17687668

[B77] Sanchuki H. B. Gravina F. Rodrigues T. E. Gerhardt E. C. Pedrosa F. O. Souza E. M. (2017). Dynamics of the *Escherichia coli* proteome in response to nitrogen starvation and entry into the stationary phase. Biochim. Biophys. Acta Protetins Proteom. 1865, 344–352. 10.1016/j.bbapap.2016.12.002 27939605

[B78] Serra D. O. Hengge R. (2014). Stress responses go three dimensional – the special order of physiological differentiation in bacterial macrocology biofilms. Environ. Microbiol. 16, 1455–1471. 10.1111/1462-2920.12483 24725389 PMC4238805

[B79] Shajani Z. Sykes M. T. Williamson J. R. (2011). Assembly of bacterial ribosomes. Annu. Rev. Biochem. 80, 501–526. 10.1146/annurev-biochem-062608-160432 21529161

[B80] Shimada T. Makinoshima H. Ogawa Y. Miki T. Maeda M. Ishihama A. (2004). Classification and strength measurement of stationary-phase promoters by use of a newly developed promoter cloning vector. J. Bacteriol. 186, 7112–7122. 10.1128/JB.186.21.7112-7122.2004 15489422 PMC523215

[B81] Shimada T. Yoshida H. Ishihama A. (2013). Involvement of cyclic AMP receptor protein in regulation of the *rmf* gene encoding the ribosome modulation factor in *Escherichia coli* . J. Bacteriol. 195, 2212–2219. 10.1128/JB.02279-12 23475967 PMC3650541

[B82] Shimada T. Shimada K. Matsui M. Kitai Y. Igarashi J. Suga H. (2014). Roles of cell division control factor SdiA: recognition of quorum sensing signals and modulation of transcription regulation targets. Genes Cells 19, 405–418. 10.1111/gtc.12139 24645791

[B83] Stewart P. S. Franklin M. J. (2008). Physiological heterogeneity in biofilms. Nat. Rev. Microbiol. 6, 199–210. 10.1038/nrmicro1838 18264116

[B84] Talukder A. Z. Iwata A. Ueda A. Ishihama A. (1999). Growth phase-dependent variation in the protein composition of *Escherichia coli* nucleoid. J. Bacteriol. 181, 6361–6370. 10515926 10.1128/jb.181.20.6361-6370.1999 PMC103771

[B85] Terui Y. Tabei Y. Akiyama M. Higashi K. Tomitori H. Yamamoto K. (2010). Ribosome modulation factor, an important protein for cell viability encoded by the polyamine modulon. J. Biol. Chem. 285, 28698–28707. 10.1074/jbc.M110.111195 20628056 PMC2937897

[B86] Trevino-Quintanilla L. G. Freyre-Gonzalez J. A. Martinez-Flores I. (2013). Anti-sigma factors in *E. coli:* common regulatory mechanisms controlling sigma factors availability. Curr. Genomics 14, 378–387. 10.2174/1389202911314060007 24396271 PMC3861889

[B87] Ueta M. Yoshida H. Wada C. Baba T. Mori H. Wada A. (2005). Ribosome binding proteins YhbH and YfiA have opposite functions during 100S formation in the stationary phase of *Escherichia coli* . Genes Cells 10, 1103–1112. 10.1111/j.1365-2443.2005.00903.x 16324148

[B88] Ueta M. Ohniwa R. L. Yoshida H. Maki Y. Wada C. Wada A. (2008). Role of HPF (hibernation promoting factor) in translational activity in *Escherichia coli* . J. Biochem. 143, 425–433. 10.1093/jb/mvm243 18174192

[B89] Ueta M. Wada C. Daifuku T. Sako Y. Bessho Y. Kitamura A. (2013). Conservation of two distinct types of 100S ribosome in bacteria. Genes Cells 18, 554–574. 10.1111/gtc.12057 23663662

[B90] Vogel H. J. Bonner D. M. (1956). Acetylornithinase of *Escherichia coli*: partical purification and some properties. J. Biol. Chem. 218, 97–106. 13278318

[B91] Wada A. Sako T. (1987). Primary structures of and genes for new ribosomal proteins A and B in *Escherichia coli* . J. Biochem. 101, 817–820. 10.1093/jb/101.3.817 3298224

[B92] Wada A. Yamazaki Y. Fujita N. Ishihama A. (1990). Structure and probable genetic location of a “ribosome modulation factor” associated with 100S ribosome in stationary-phase *Escherichia coli* cells. Proc. Natl. Acad. Sci. U.S.A. 87, 2657–2661. 10.1073/pnas.87.7.2657 2181444 PMC53749

[B93] Wada A. Igarashi K. Yoshimura S. Aimoto S. Ishihama A. (1995). Ribosome modulation factor: stationary growth phase-specific inhibitor of ribosome functions from *Escherichia coli* . Biochem. Biophys. Res. Commun. 214, 410–417. 10.1006/bbrc.1995.2302 7677746

[B94] Wada A. (1986a). Analysis of *Escherichia coli* ribosomal proteins by an improved two dimensional gel electrophoresis. I. Detection of four new proteins. J. Biochem. (Tokyo) 100, 1583–1594. 10.1093/oxfordjournals.jbchem.a121866 3553168

[B95] Wada A. (1986b). Analysis of *Escherichia coli* ribosomal proteins by an improved two dimensional gel electrophoresis. II. Characterization of four new proteins. J. Biochem. (Tokyo) 100, 1595–1605. 10.1093/oxfordjournals.jbchem.a121867 3553169

[B96] Wada A. (1998). Growth phase coupled modulation of *Escherichia coli* ribosomes. Genes Cells 3, 203–208. 10.1046/j.1365-2443.1998.00187.x 9663655

[B97] Westblade L. F. Ilag L. L. Powell A. K. Kolb A. Robinson C. V. Busby S. J. W. (2004). Studies of the Escherichia coli Rsd-sigma 70 complex. J. Mol. Biol. 335, 685–692. 10.1016/j.jmb.2003.11.004 14687566

[B98] Yamagishi M. Matsushima H. Wada A. Sakagami M. Fujita N. Ishihama A. (1993). Regulation of the *Escherichia coli rmf* gene encoding ribosome modulation factor (RMF): Growth phase- and growth rate-control. EMBO J. 12, 625–630. 10.1002/j.1460-2075.1993.tb05695.x 8440252 PMC413246

[B99] Yamamoto N. Nakahigashi K. Nakamichi T. Yoshino M. Takai Y. Touda Y. (2009). Update on the Keio collection of *Escherichia coli* single-gene deletion mutants. Mol. Syst. Biol. 5, 335. 10.1038/msb.2009.92 20029369 PMC2824493

[B100] Yoshida H. Wada A. (2014). The 100S ribosome: ribosomal hibernation induced by stress. Wiley Interdiscip. Rev. RNA 2014, 723–732. 10.1002/wrna.1242 24944100

[B101] Yoshida H. Maki Y. Kato H. Fujisawa H. Izutsu K. Wada C. (2002). The ribosome modulation factor (RMF) binding site on the 100S ribosome of *Escherichia coli* . J. Biochem. 132, 983–989. 10.1093/oxfordjournals.jbchem a003313. 12473202 10.1093/oxfordjournals.jbchem.a003313

[B102] Yoshida H. Yamamoto H. Uchiumi T. Wada A. (2004). RMF inactivates ribosomes by covering the peptidyl transferase centre and entrance of peptide exit tunnel. Genes Cells 9, 271–278. 10.1111/j.1356-9597.2004.00723.x 15066119

[B103] Yoshida H. Shimada T. Ishihama A. (2018). Coordinated hibernation of transcriptional and translational apparatus expression during growth transition of *Escherichia coli* into stationary phase. mSystems 3, e00057–e00018. 10.1128/mSystems 00057-18. 30225374 10.1128/mSystems.00057-18 PMC6134199

[B104] Yuan A. H. Gregory B. D. Sharp J. S. McCleary K. D. Dove S. L. Hochschild A. (2008). Rsd family proteins make simultaneous interactions with regions 2 and 4 of the primary sigma factor. Mol. Microbiol. 70, 1136–1151. 10.1111/j.1365-2958.2008.06462.x 18826409 PMC2581641

[B105] Zengel J. M. Lindahl L. (1994). Diverse mechanisms for regulating ribosomal protein synthesis in *Escherichia coli* . Prog. Nucleic Acid Res. Mol. Biol. 47, 331–370. 10.1016/s0079-6603(08)60256-1 7517053

[B106] Zinser E. R. Kolter R. (2004). *Escherichia coli* evolution during stationary phase. Res. Microbiol. 155, 328–336. 10.1016/j.resmic.2004.01.014 15207864

