# Supplementary material for: Coordinated Regulation of Rsd and RMF for Simultaneous Hibernation of Transcription Apparatus and Translation Machinery in Stationary-Phase Escherichia coli
Source: Front Genet. 2019 Dec 4;10:1153. doi: 10.3389/fgene.2019.01153 (PMC6904343; doi:10.3389/fgene.2019.01153)
Supplement: Supplemental Figure S1 — RFHR 2D gel patterns of E. coli proteins. E. coli K-12 AD202 was grown at 37 °C in minimal medium E containing 2% peptone. Cell lysates were prepared as described in text, and fractionated by centrifugation into CD (Insoluble cell debris), CE (supernatant cell extract), CR (crude ribosome), and PRS (post ribosomal supernatant) fractions. Soluble proteins of the CR [A], PRS [B], basic CD [C] and acidic CD [C] were subjected to RFHR (radical free and highly reducing) method of 2D gel analysis (Wada 1986a; Wada, 1986b). Gels were stained with CBB. [file DataSheet_1.pdf]

Supplemental Table S1

Proteins Expressed During Prolonged Culture of *Escherichia coli* K-12

PRS (Post Ribosomal Supernatant) fraction

2D spot	*Gene*	pI/Size (aa)	Function	Max stage
PRS01	*X*			Day 7
RPS02	*oppA*	6.05/543	periplasmic oligopeptide transporter protein	Day 7 and 8
PRS03	*guaB*	6.02/486	Inosine 5'-monophosphate dehydrogenase	Day 8
PRS04	*ldtE*	9.42/334	L,D-transpeptidase	10 h
RPS05	*mdh*	5.61/312	malate dehydrogenase	Day 8
PRS06	*gatY*	5.87/284	tagarose-1,6-dibphosphate aldolase	Day 2
PRS07	*hchA*	5.63/283	protein/nucleic acid deglycase	Day 8
RPS08	*rbsB*	6.85/296	periplasmic ribose transperter protein	Day 6
PRS09	*gloC*	4.95/215	hydroxyacylglutathione hydrolase	Day 3
PRS10	*modA*	7.81/257	periplasmic molybdate transporter protein	Day 7
RPS11	*modA*	7.81/257	periplasmic molybdate transporter protein	Day 3
PRS12	*nfuA*	4.52/191	iron-sulfur cluster carrier protein	Late-log (3 h)
PRS13	*X*			Late-log (3 h)
PRS14	*slyD*	4.86/196	FKBP-type pepridyl-prolyl cis-trans isomerase	Late-log (3 h)
RPS15	*rbsD*	5.93/139	D-ribose pyranase	Late-log (3 h)
PRS16	*uspG*	6.03/142	universal stress protein G	Day 1
RPS17	*uspD*	6.37/142	universal stress protein D	Day 1
PRS18	*hipA*	8.26/440	serine/threonine kinase Hipa	Late-log (5 h)
PRS19	*X*			Day 5
RPS20	*hdeA*	5.06/110	periplasmic acid stress chaperone HdeA	Late-log (5 and 6 h)
PRS21	*ralA*	6.19/113	stationary-phase translation inhibitor/ribosome stability factor	Day 7
PRS22	*zapB*	4.69/81	cell division factor ZapB	Late-log (3 and 5 h)
RPS23	*maoP*	6.09/112	macrodomain Ori protein	Log
PRS24	*yccJ*	4.70/75	PF13993 family protein YccJ	Late-log (5 h)
PRS25	*cspC*	6.54/69	cold-shock stress protein CspC	Late-log (3 h)
RPS26	*cspE*	8.09/69	transcription antiterminator/RNA stability regulator CspE	Late-log (3 h)
PRS27	*X*			Late-log (2 h)
PRS28	*X*			Day 2
RPS29	*yibJ*	5.00/?	RHA domain-containing protein YibJ	10 h
RPS30	*X*			Day 7
PRS31	*ydcH*	9.30/74	uncharacterized protein	Day 2

CD (insoluble cell debris) fraction

2D spot	*Gene*	pI/Size (aa)	Function	Max stage
CD01	*ompC*	4.58/367	outer membrane protein C pore for passive difusion	Day 4
CD02	*gatY*	5.87/284	tagarose-1,6-dibphosphate aldolase	Day 4
CD03	*rtsB*	6.85/296	periplasmic ribose transporter protein	Day 5
CD04	*X*			Day 5
CD05	*slyD*	1.86/196	FKBP-type peptidyl-prolyl cis-trans isomerase SlyD	Day 3
CD06	*X*			Late-log (3 h)
CD07	*dps*	5.70/167	stationary-phase nucleoid protein/ison sequester protein	10 h
CD08	*X*			Log
CD09	*kbp*	5.67/149	K+ binding protein	Day 6
CD10	*X*			Day 5 and 7
CD11	*uspG*	6.03/142	universal stress protein G	Log
CD12	*uspD*	6.37/142	universal stress protein D	Day 3 and 7
CD13	*stpA*	7.95/134	nucleoid protein StpA with RNA chaperone activiry	Log
CD14	*X*			Day 6
CD15	*elaB*	5.35/101	tail-anchored inner membrane protein	10 h and Day 1
CD16	*X*			Log
CD17	*X*			Log
CD18	*X*			Late-log (5 h)
CD19	*raiA*	6.19/113	stationary-phase translation inhibitor/ribosome stability factor	10 h
CD20	*X*			Late-log (5 hr)
CD21	*zapB*	4.69/81	cell division factor ZapB	Day 8
CD22	*X*			Day 8

CD23	*ydcH*	9.30/74	uncharacterized protein	Day 7 and 8
CD24	*yggX*	5.91/91	Fe2+-tracking protein	Day 5
CD25	*yeeX*	9.30/109	DUF496 domain-containing protein	Late-log (3 h)
CD26	*X*			Log
CD27	*yqjD*	9.06/101	ribosome- and membrane-associated DUF-domain protein	Day 2
CD28	*yibJ*	5.00/?	RHA domain-containing protein YibJ	Day 3
CD29	*ydcH*	9.30/74	uncharacterized protein	Day 3 and 4
CD30	*ymdF*	9.87/57	stress-induced acidphilic repreak motifs-containing protein	10 h

CR (Crude Ribosome) fraction

2D spot	*Gene*	pI/Size (aa)	Function	Max stage
CR01	*rmf*	10.86/55	ribosome modulation factor	Day 1 and 2
CR02	*raiA*	6.19/113	stationary-phase translation inhibitor/risobome stability factor	Day 2 and 3
CR03	*hpf*	6.50/95	ribosome hibernation-promoting factor	10 h
CR04	*sra*	11.04/45	30S ribosomal protein S22	10 h

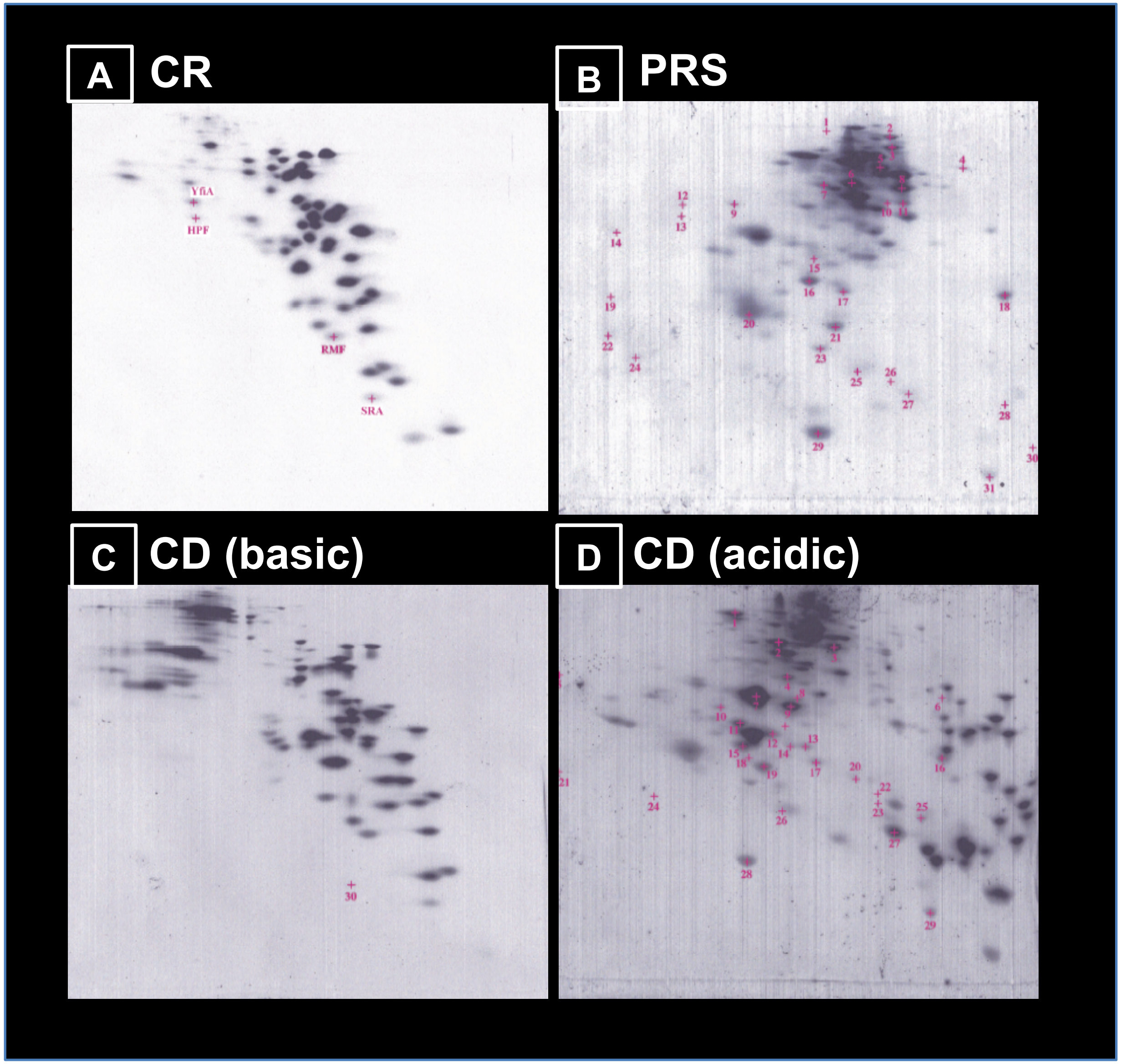

Yoshida et al. (Figure S1)

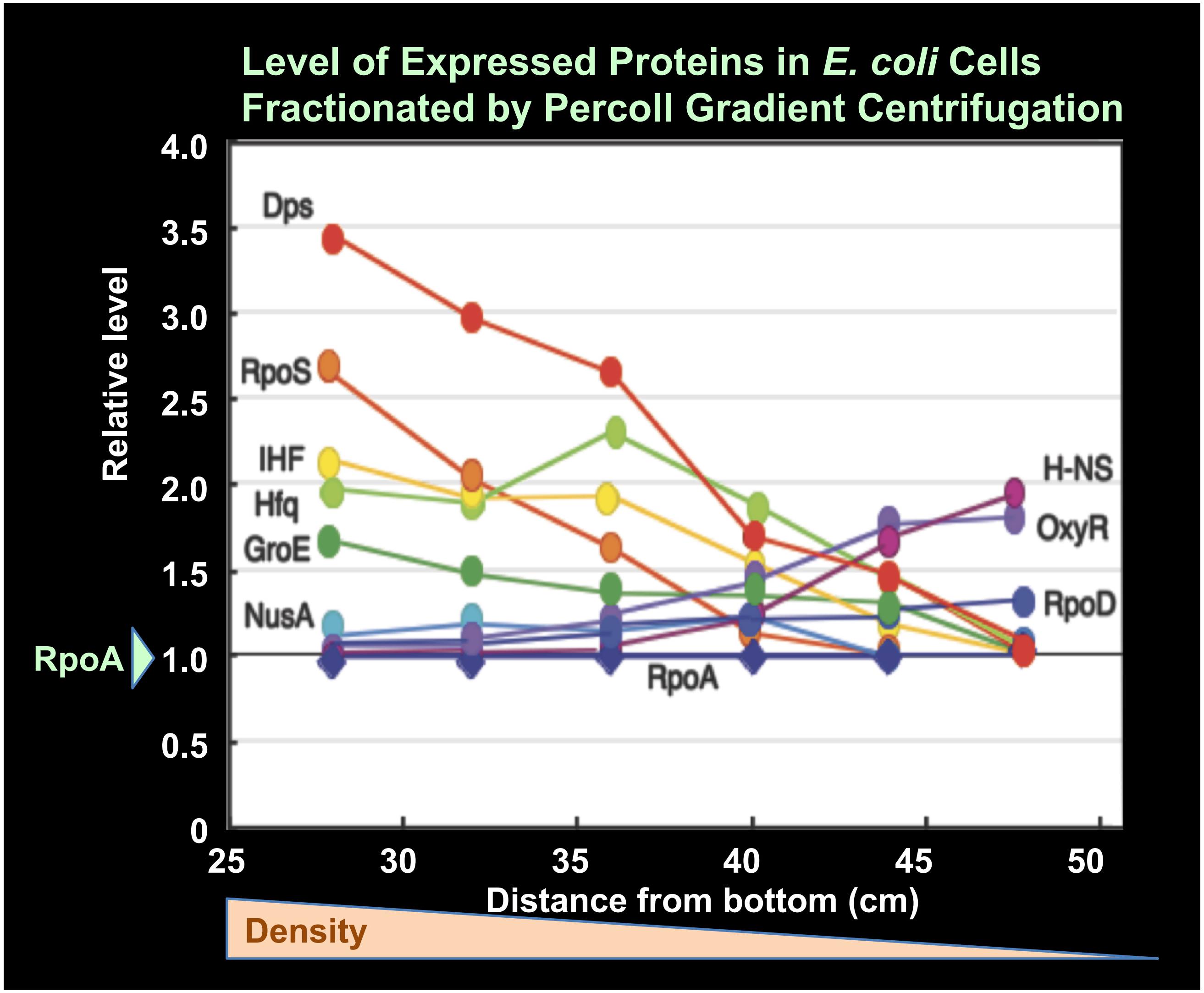

Yoshida et al. (Figure S2)

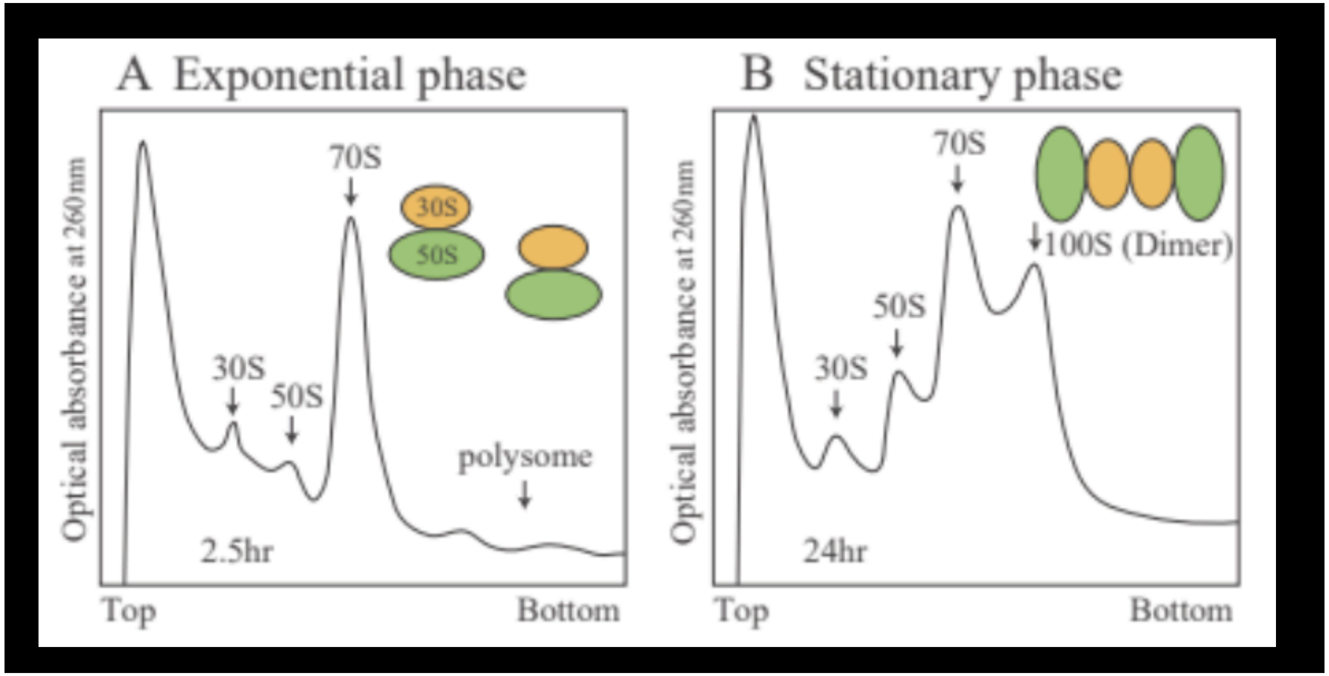

Yoshida et al. (Figure S3)
